# Genetic Dissection of the Function of Hindbrain Axonal Commissures

**DOI:** 10.1371/journal.pbio.1000325

**Published:** 2010-03-09

**Authors:** Nicolas Renier, Martijn Schonewille, Fabrice Giraudet, Aleksandra Badura, Marc Tessier-Lavigne, Paul Avan, Chris I. De Zeeuw, Alain Chédotal

**Affiliations:** 1 INSERM, U968, Paris, F-75012, France; 2 UPMC Univ Paris 06, UMR_S 968, Institut de la Vision, Paris, F-75012, France; 3 CNRS, UMR_7210, Paris, F-75012, France; 4 Department of Neuroscience, Erasmus MC, Rotterdam, The Netherlands; 5 Laboratory of Sensory Biophysics, School of Medicine, University of Auvergne, Clermont-Ferrand, France; 6 Genentech, South San Francisco, California, United States of America; 7 Netherlands Institute for Neuroscience, Royal Dutch Academy of Arts and Sciences (KNAW), Amsterdam, The Netherlands; University of Cambridge, United Kingdom

## Abstract

In Bilateria, many axons cross the midline of the central nervous system, forming well-defined commissures. Whereas in mammals the functions of commissures in the forebrain and in the visual system are well established, functions at other axial levels are less clearly understood. Here, we have dissected the function of several hindbrain commissures using genetic methods. By taking advantage of multiple Cre transgenic lines, we have induced site-specific deletions of the Robo3 receptor. These lines developed with the disruption of specific commissures in the sensory, motor, and sensorimotor systems, resulting in severe and permanent functional deficits. We show that mice with severely reduced commissures in rhombomeres 5 and 3 have abnormal lateral eye movements and auditory brainstem responses, respectively, whereas mice with a primarily uncrossed climbing fiber/Purkinje cell projection are strongly ataxic. Surprisingly, although rerouted axons remain ipsilateral, they still project to their appropriate neuronal targets. Moreover, some *Cre;Robo3* lines represent potential models that can be used to study human syndromes, including horizontal gaze palsy with progressive scoliosis (HGPPS). To our knowledge, this study is one of the first to link defects in commissural axon guidance with specific cellular and behavioral phenotypes.

## Abstract 2

The Robo3 receptor controls midline crossing by axons. Deleting Robo3 in specific commissural neurons with a conditional knockout reveals their contribution to sensory and motor integration, and models human neurological conditions.

## Author Summary

Coordination of the left and right sides of the body requires the action of neurons whose axons cross the nervous system midline. The precise contributions of “commissural” neurons to sensory and motor functions remain poorly understood. To probe these crossing circuits, we took advantage of the recent finding that the Robo3 axon guidance receptor is required for midline crossing by axons at most axial levels. A Robo3 conditional knockout mouse line was generated, allowing Robo3 to be deleted in selective neuronal populations. This led to disruption of specific commissures in the sensory, motor, and sensorimotor systems, and resulted in severe but specific functional deficits. Surprisingly, although rerouted axons do not cross the midline, they still project to their appropriate neuronal targets, suggesting that midline crossing is not required to complete the axonal guidance program of those neurons. Moreover, some of the mouse lines represent good models for human syndromes, including horizontal gaze palsy with progressive scoliosis (HGPPS), which is characterized by deficits in coordinated eye movements. This study links defects in commissural axon guidance with specific and dramatic behavioral phenotypes.

## Introduction

At all levels of vertebrate nervous systems, axons cross the midline to form commissural projections [1],[2]. In the visual system and the corpus callosum in the neocortex, the physiological importance of brain commissures is well established in large part due to the “split-brain” studies of Roger Sperry [3] and others. However, the function of commissural projections in the hindbrain and spinal cord has been more difficult to assess. Surgically ablating specific commissures in the hindbrain has proved to be difficult [4]. In addition, so far, no mutant has been created in which these connections are disrupted in a region-specific manner. Therefore, the function of specific commissures in a variety of hindbrain systems remains uncertain.

By contrast, considerable progress has been made over the past two decades in understanding how commissures form during embryonic development. Genetic screens in *Drosophila*, *Caenorhabditis elegans*, and mice, as well as biochemical approaches in rodents, have shown that the molecular mechanisms regulating commissure development are highly conserved in evolution [5] and involve axon guidance molecules, including Netrins, Slits, Semaphorins, Ephrins, Morphogens, and IgCAMs [6]. Their functions are tightly regulated by ECM molecules, cytosolic second messengers, receptor dimerization, and protein degradation in addition to transcriptional and posttranslational modifications [6],[7].

To broaden our understanding of the function of hindbrain commissures, we undertook to genetically rewire the hindbrain by forcing select commissural tracts to remain ipsilateral, and then examined the cellular and behavioral consequence of these manipulations. We focused on Robo3, a vertebrate roundabout receptor involved in the formation of hindbrain and spinal cord commissures [8],[9]. Robo3, also called *Rig-1* (retinoblastoma inhibiting gene 1), was isolated as the product of a gene up-regulated in retinoblastoma-deficient mice [10]. In humans, mutations in *ROBO3* cause a rare syndrome called horizontal gaze palsy with progressive scoliosis (HGPPS) [11],[12]. HGPPS patients have both an uncrossed pyramidal tract and dorsal column-medial lemniscus as well as severe scoliosis. Despite defects in conjugate horizontal eye movements [13], they are reasonably coordinated and do not present other obvious neurological deficits. The cause of horizontal gaze palsy, one of two signature traits of HGPPS syndrome, is still uncertain [11]. In addition, a subset of HGPPS patients show an unexplained asymmetry of brainstem auditory evoked potentials and activation of their auditory cortex [13],[14].

Studies in mice show that in hindbrain and spinal cord, Robo3 is required for commissural axons to cross the midline and that commissural neurons, deficient in Robo3, extend their axons ipsilaterally [8],[9]. The genetic manipulation of Robo3 could thus provide effective means to characterize roles for hindbrain commissures and lead to understanding the etiology of the HGPPS syndrome. Because existing Robo3 knockout mice die perinatally, precluding behavioral analysis [9], we have undertaken the construction of a conditional allele of Robo3 and show that viable animals lacking specific commissures can be obtained.

## Results

### A Reduced Internuclear Commissure in *Robo3*-Deficient Mice Induces Abnormal Horizontal Compensatory Eye Movements

**Figure 1:**
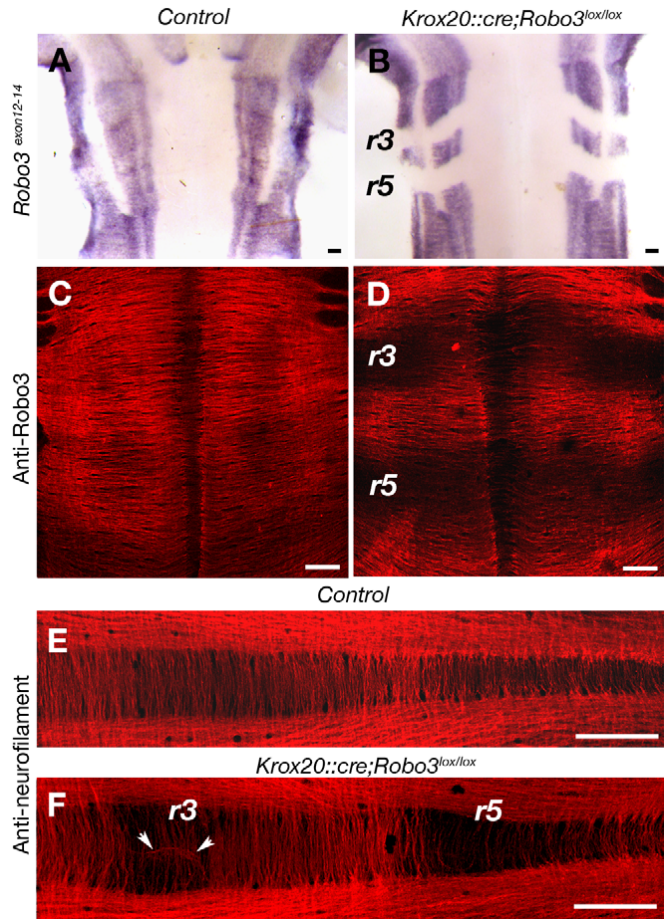
Rhombomere-specific deletion of Robo3. Whole-mount control (A, C, and E) or *Krox20::cre;Robo3^lox/lox^* (B, D, and F) E12 embryos hybridized with a *Robo3* riboprobe covering exons 12–14 (A and B) or immunostained with anti-Robo3 (C and D) or anti-neurofilament (E and F) antibodies. (B) In *Krox20::cre;Robo3^lox/lox^* embryos, *Robo3^exon12-14^* transcripts are not expressed in rhombomeres 3 (r3) and 5 (r5). (C and D) Likewise, there is a severe reduction of Robo3 immunoreactive commissural axons in r3 and r5. (E and F) Anti-neurofilament immunostaining confirms the strong reduction of commissures in r3 and r5. The arrowheads in (F) indicate axons that abnormally follow the midline. Scale bars represent 100 µm.

**Figure 2:**
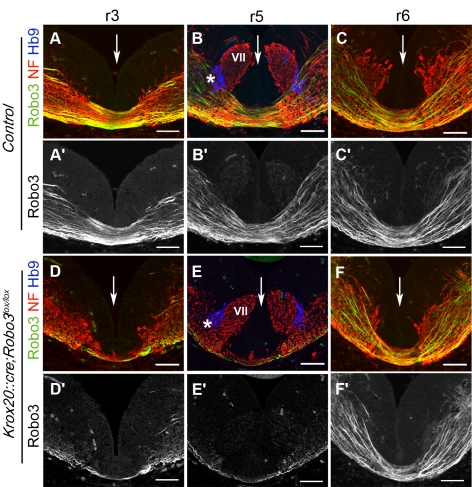
Rhombomere-specific deletion of commissures. Coronal sections at the level of r3, r5, and r6 of E11 control (A–C′) or *Krox20::cre;Robo3^lox/lox^* (D–F′) embryos immunostained with antibodies against Robo3, neurofilament, and Hb9. The midline is indicated by an arrow, and the Hb9-positive abducens motor neurons by an asterisk. In r3 and r5, there is an almost complete absence of Robo3 and neurofilament-positive commissural axons in *Krox20::cre;Robo3^lox/lox^* (D–E′) compared to controls (A–B′). The density of neurofilament-positive axons is also strongly reduced. By contrast, there is no obvious reduction of commissural axons or Robo3 expression in r6 (C, C′, F, and F′). Note that at E11, abducens shape and position are not altered in mutants compared to controls (see Figure 3 for later stages). VII, migrating facial neurons and facial nerve. Scale bars represent 60 µm.

In vertebrates, lateral eye movements are controlled by two pairs of cranial motor nuclei, the abducens (VI) and the oculomotor (III) nuclei, which project ipsilaterally to the lateral and medial rectus muscles, respectively [15] (Figure S1). Conjugate lateral eye movement requires bilateral coordination of those muscles, which relies in part on abducens interneurons that project to the contralateral oculomotor nucleus, forming the abducens-oculomotor (III) internuclear commissural projection (Figure S1). Furthermore, in functional magnetic resonance imaging studies of HGPPS patients, nucleus VI is hypoplastic, whereas cranial nerves III and VI are present bilaterally, suggesting that this palsy may result from defects in commissural systems [11]. We, therefore, analyzed the oculomotor system in the existing *Robo3*-null mice (*Robo3^−/−^*) [9] as well as in a *Robo3*-conditional mouse that lacked *Robo3* in rhombomere 5 (r5), the rhombomere from which abducens neurons are derived. The latter mice (*Krox20::cre;Robo3^lox/lox^*) were generated by creating *Robo3^lox/lox^* mice (Figure S2) and crossing them with *Krox20::cre* knock-in mice that express Cre recombinase in r3 and r5 [16]. Upon Cre-mediated excision of exons 12–14, a truncated Robo3 protein is produced, which lacks transmembrane and cytoplasmic domains, thereby disabling it as a receptor. R3-r5–specific *Robo3* inactivation was confirmed by performing whole-mount labeling with a *Robo3* exon-specific probe and Robo3 antibody on embryonic day (E)12 embryos (Figure 1). As expected, severe reduction of commissural projections in r3 and r5 was observed in whole-mount (Figure 1; *n* = 2/2) and coronal sections of *Krox20::cre;Robo3^lox/lox^* E11–E12 embryos labeled with anti-neurofilament and anti-Robo3 antibodies (Figure 2 *n* = 3/3). By contrast, many commissural axons were still observed in adjacent rhombomeres (Figure 2). A few Robo3-positive axons still crossed the midline at the r3 and r5 level in *Krox20::cre;Robo3^lox/lox^* embryos. These fibers most likely originate in commissural neurons located outside of r3 and r5 [17] (see Discussion). In *Krox20::cre;Robo3^lox/lox^* embryos, the expression of the known floor plate–derived axon guidance molecules, *Netrin1*, *Sonic Hedgehog* (*Shh*), *Slit1*, *Slit2*, and *Slit3* was not perturbed in r3 and r5 (Figure S3; *n* = 2/2).

**Figure 3:**
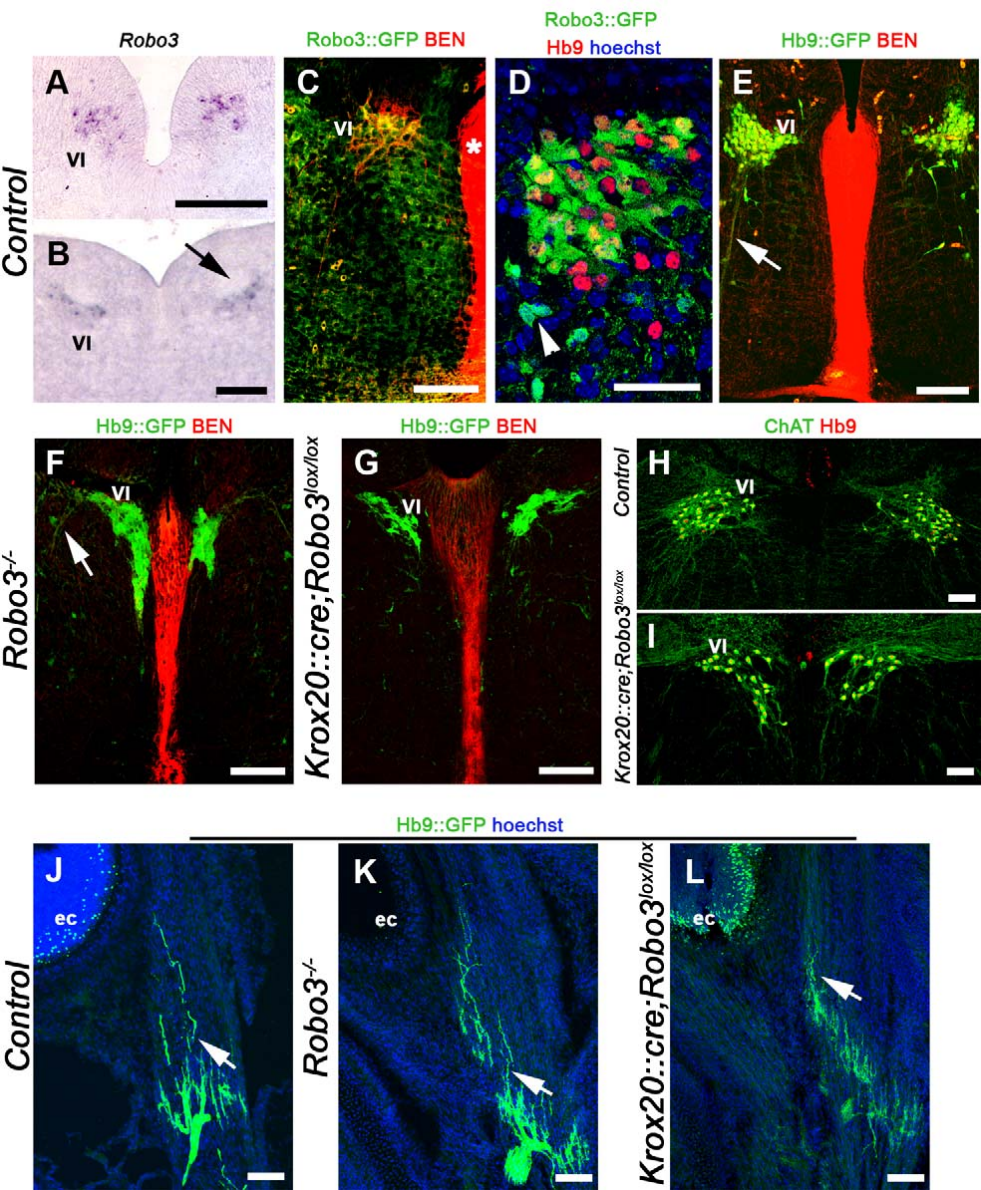
Normal projection of abducens motor axons in Robo3-deficient mice. (A to I) show coronal hindbrain sections at the level of the abducens nucleus. (A and B) *Robo3* transcripts are detected in the abducens nuclei of E12 (A) and E14 (B) embryos. The arrow in (B) points to the facial nerve. (C) abducens neurons coexpress BEN and GFP in *Robo3^+/−^* E14 embryo. The floor plate (asterisk) also expresses BEN. (D) Hb9/GFP double labeling in E15 *Robo3^+/−^* embryo. Note that some GFP+ cells (arrowhead) do not express Hb9. (E) abducens neurons (VI) and axons (arrow) express GFP in E14 *Hb9::GFP* transgenic embryo. (F–I) The abducens nucleus has an abnormal shape and is closer to the floor plate in *Robo3^−/−^* (F) and *Krox20::cre;Robo3^lox/lox^* (G) E14 embryos (compare with [E]). The arrow in (F) points to abducens axons. (H and I) This abnormal shape and position are also observed in adult animals with ChAT/Hb9 double immunostaining. (J to L) Coronal sections of P0 mouse head at eye cup (ec) level. GFP-positive abducens axons (arrows) still contact the lateral rectus muscle in *Robo3*-null embryo (K) and *Krox20::cre;Robo3^lox/lox^* mutant (L). Scale bars represent 100 µm, except in (D), where it indicates 50 µm.

Unlike *Robo3^−/−^* null mice, which die at birth [9], *Krox20::cre;Robo3^lox/lox^* mice are viable (*n* = 29/29) and do not show any obvious behavioral defects compared to *Robo3^lox/lox^* or wild-type controls. To find out whether Robo3 is expressed in the abducens nuclei, we performed in situ hybridization and analyzed GFP immunostaining in *Robo3^+/−^* heterozygous embryos in which GFP was knocked-in to the *Robo3* locus (*n* = 3/3) [9]. We found that from E12 to E14, *Robo3* is expressed by a subset of neurons in the abducens nucleus, but not in other cranial motor nuclei (Figure 3A and 3B). Accordingly, in *Robo3^+/−^* embryos, GFP was expressed by Hb9 and BEN/SC1 immunopositive motor neurons (Figure 3C and 3D; *n* = 2/2). However, it was also detected in Hb9-negative neurons (Figure 3D), most likely corresponding to commissural interneurons projecting to the contralateral oculomotor nucleus. To discern abducens axons from other oculomotor axons and follow their trajectory, we next crossed *Krox20::cre;Robo3^lox/lox^* mice to *Hb9::GFP* transgenic mice [18], which, in the hindbrain, selectively express GFP in the VI and hypoglossus (XII) somatic motor neurons (Figure 3E). Analysis of Hb9::GFP labeling and immunostaining or in situ hybridization with motor neuron markers (Hb9, BEN/SC1, Islet-1, ChAT, CGRP) revealed that, from E12, the shape and mediolateral position of the abducens motor nuclei were slightly altered in *Robo3^−/−^* (*n* = 16/16) and *Krox20::cre;Robo3^lox/lox^* mice (*n* = 9/9; Figure 3F–3I and Figure S4). However, abducens motor axons still selectively projected to the lateral rectus muscle (*n* = 3/3 in *Robo3^−/−^* and *n* = 2/2 *in Krox20::cre;Robo3^lox/lox^*; Figure 3J–3L). GFP-positive abducens axons were never found to cross the midline, suggesting that, as in controls, the projection remains ipsilateral. In addition, the numbers of Hb9-positive VI neurons were similar in *Krox20::cre;Robo3^lox/lox^* and control mice (211±28 and 205±19 neurons per nucleus, respectively, *p* = 0.8, n = 4 animals for each genotype; Student *t*-test with Mann-Whitney hypothesis, see Materials and Methods).

**Figure 4:**
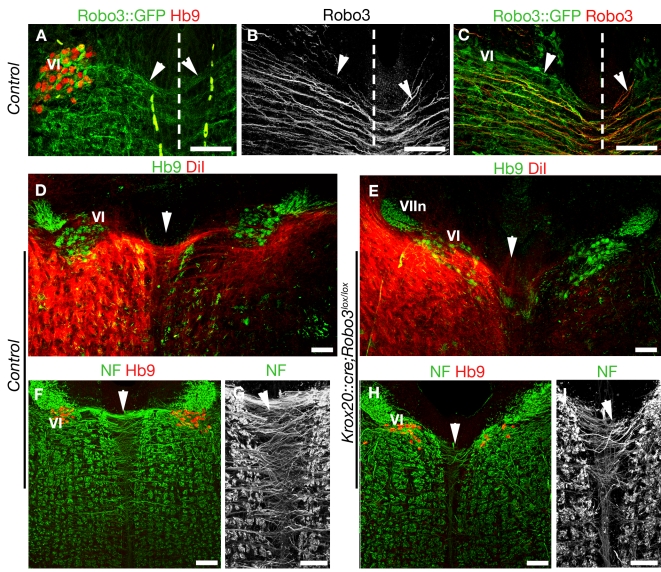
Reduced internuclear commissure in Robo3 knockout mice. (A to I) show coronal hindbrain sections at the level of the abducens nucleus, visualized by Hb9 immunostaining (in A, D, E, F, and H). (A, B, and C) illustrate the projection of abducens axons (arrowheads) across the midline (dashed line) in *Robo3^+/−^* E13 embryos. Some GFP+ axons originate from the abducens nucleus (VI) and are immunoreactive for Robo3 (B and C). (D) The internuclear commissure (arrowhead) is also observed in P0 controls, following DiI injection at the level of the oculomotor nucleus III. (E) This commissure is almost completely absent in P0 *Krox20::cre;Robo3^lox/lox^* mice (arrowhead). (VIIn): Genu of facial nerve. (F to I) At E15, many neurofilament+ axons cross the midline at the VI level (arrowheads) in control embryo (F and G), whereas they are rare in *Krox20::cre;Robo3^lox/lox^* embryo (H and I). Scale bars represent 100 µm, except in (G and I), where they indicate 50 µm.

Commissural axons that crossed the midline at the level of the abducens nucleus express Robo3 in E13 *Robo3^+/−^* embryos (Figure 4A–4C; *n* = 3/3). To determine the need for Robo3, we studied the abducens-oculomotor (III) internuclear commissural projection by injecting 1,1′ dioctadecyl-3,3,3′,3′ tetramethylindocarbocyanine (DiI) tracer unilaterally at the level of the III nucleus. In control newborns (*n* = 6/6), this injection resulted in retrograde labeling of many neurons at the level of the contralateral abducens nucleus and of their axons crossing the floor plate (Figure 4 and Figure S5). On the other hand, in *Robo3^−/−^* (*n* = 3/3; Figure S5) and *Krox20::cre;Robo3^lox/lox^* (*n* = 3/3; Figure 4E) newborn mice, contralateral labeling was virtually absent, whereas some neurons were retrogradely labeled in nucleus VI on the ipsilateral side. In addition, in *Robo3* ^−/−^ embryos (*n* = 3/3), no GFP-positive axons were found to cross the midline at the level of the abducens nuclei at E14 (unpublished data), and in E15 *Krox20::cre;Robo3^lox/lox^* embryos (*n* = 4/4), there was a severe reduction of neurofilament-positive axons crossing the midline at the level of the abducens nucleus (Figure 4F–4I). Together, these results indicate that deletion of Robo3 either globally or specifically in r5 neurons results in loss of the abducens-oculomotor (III) internuclear commissural projection without markedly affecting the pathfinding of abducens motor axons.

**Figure 5:**
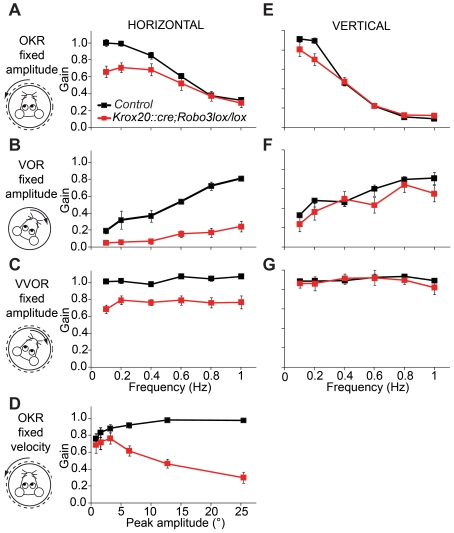
Impaired horizontal compensatory eye movements in *Krox20::cre;Robo3^lox/lox^* mice. (A) During optokinetic stimulation horizontal gains are reduced most prominently at the lower frequencies in in *Krox20::cre;Robo3^lox/lox^* mice (*p* = 0.043 ANOVA; *n* = 6 versus *n* = 4 for controls; see Table S1). (B) At higher frequencies, the VOR is severely impaired (*p* = 0.001 versus control mice curve; ANOVA for repeated measurements; Table S1), confirming the importance of the commissural connections in large-amplitude eye movements. (C) When horizontal visual and vestibular inputs are combined in the VVOR (visual vestibulo-ocular reflex), it results in lower gains over the entire range of frequencies tested (*p* = 0.004 versus control mice curve; ANOVA for repeated measurements). (D) OKR deficits are strongly correlated to the amplitude of stimulation. (E–G) In marked contrast, in the vertical plane, no significant differences were observed in OKR (E), VOR (F), or VVOR (G), supporting the concept that primarily horizontal eye movements require the presence of commissural connections (see Table S1 for all statistics). Error bars indicate standard error of the mean. Results were obtained from four control and six *Krox20::cre;Robo3^lox/lox^ mice*.

To analyze the functional consequence of the lack of the VI-III internuclear commissure we studied compensatory eye movements in adult *Krox20::cre;Robo3^lox/lox^* mice. The animals were provided with a pedestal and subjected to optokinetic and/or vestibular stimulation in both the horizontal and vertical plane (for Materials and Methods, see [19], and Text S1 and Table S1 for statistics). All three reflexes in the horizontal plane, including the horizontal optokinetic reflex (OKR), the horizontal vestibulo-ocular reflex (VOR) as well as the horizontal visual VOR (VVOR), showed striking changes in the mutants (*n* = 6) compared with control (*n* = 4) littermates (Figure 5A–5D). Conversely, all eye movement reflexes in the vertical plane were unaffected (Figure 5E–5G). During OKR, the impairments in the *Krox20::cre;Robo3^lox/lox^* mice appeared to occur predominantly in the low-frequency range, whereas during VOR, they were most prominent at higher frequencies. These frequency-specific deficits reflect the potential role of internuclear commissural connections in high-amplitude eye movements, because OKR and VOR evoke high gain values at the lower and higher frequencies, respectively [20]. This view is further supported by the fact that the gain of eye movements in the *Krox20::cre;Robo3^lox/lox^* mice decreased in a virtually linear manner with respect to stimulus amplitude at higher amplitude values (e.g., for OKR, see Figure 5D; *p*<0.001 versus control mice curve; ANOVA for repeated measurements). As a consequence, the amplitude of the horizontal VVOR, which is high over the entire frequency range because of its combined OKR and VOR nature, was significantly affected in mutants at both the lower and higher frequency ranges. The finding that all horizontal reflexes, but no vertical reflexes, were disrupted suggests that the abducens-oculomotor internuclear commissure was selectively affected.

### Ipsilateral aVCN-MNTB Projections and Impaired Auditory Brainstem Responses in *Krox20;Robo3lox* Mice

**Figure 6:**
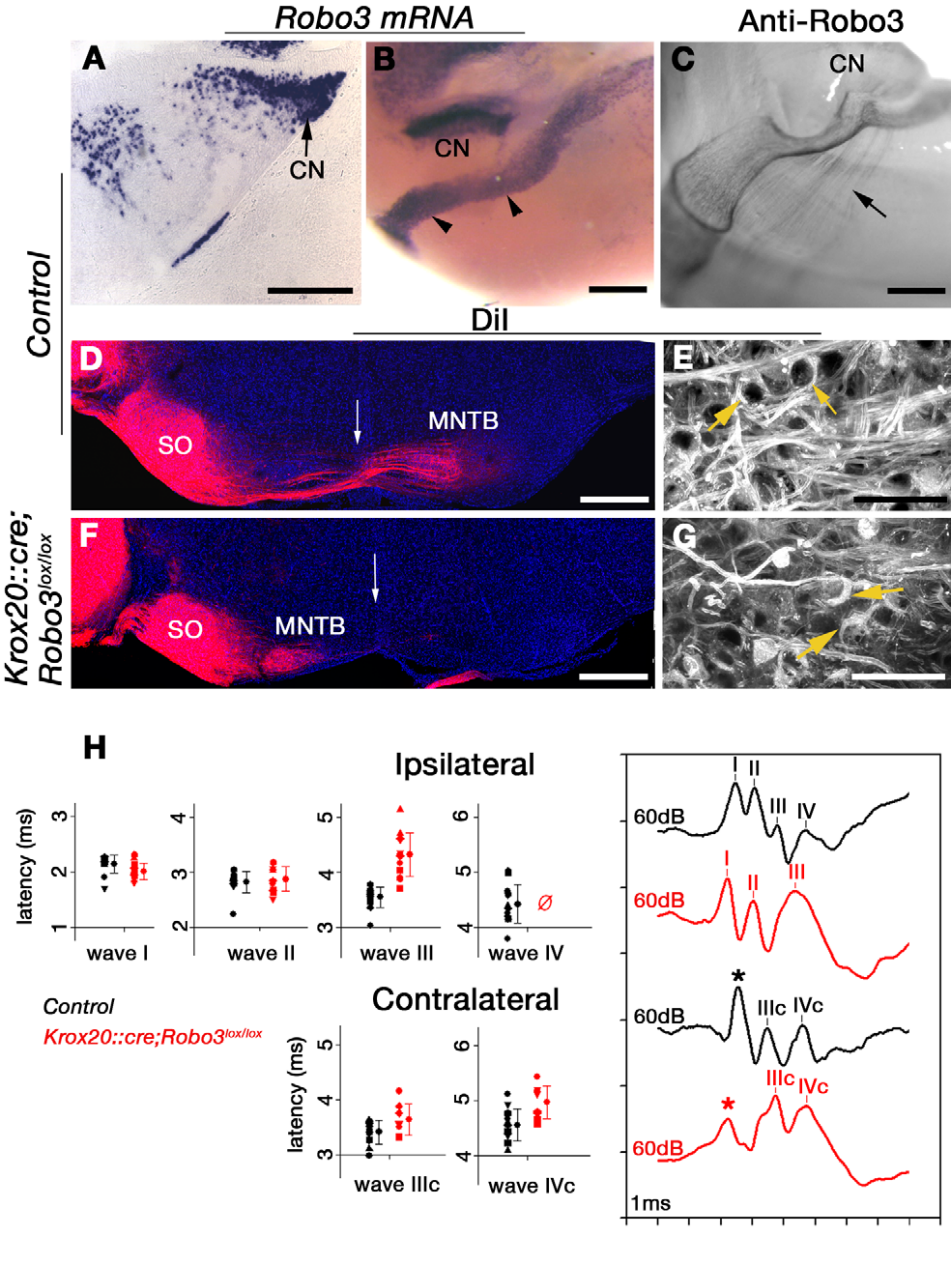
Uncrossed aVCN-MNTB projections and abnormal ABRs in *Krox20::cre;Robo3^lox/lox^* mice. (A to C) Robo3 is expressed by neurons of the cochlear nucleus (CN). Coronal sections of a E14 embryo (A) or side view of whole-mount E14 embryos (B and C) hybridized with a *Robo3* probe (A and B) or labeled with anti-Robo3 antibodies (C). The arrowheads in (B) indicate migrating pontine neurons. The arrow in (C) points to cochlear axons. (D–G) Coronal sections of P12 mice injected with DiI in the cochlear nucleus (Hoechst counterstaining). In control (D and E), DiI-labeled axons end in the ipsilateral superior olive (SO) and the contralateral MNTB, whereas in *Krox20::cre;Robo3^lox/lox^* mutant (F and G), all axons project ipsilaterally. The arrow in (D and F) indicates the midline. (E and G) are high magnification pictures of the MNTB showing DiI-labeled calyces of Held (arrows). (H) ABRs collected from the ipsilateral or contralateral mastoid electrode in response to 60 dB SPL clicks. Ipsilaterally, only three waves were observed in mutant instead of four in controls. Mutant wave III exhibited a mean latency of about 4.3 ms, much longer than that of control wave III (3.6 ms; *n* = 13 controls versus *n* = 12 mutants, average latency difference 0.77 ms for wave III; *p*<0.0001; unpaired Student *t*-test), yet too short for matching the latency of wave IV in controls. Contralaterally, in mutants, the mean latency of IVc was 0.42 ms longer than in controls (*n* = 12 controls versus *n* = 8 mutants; *p* = 0.006; unpaired Student *t*-test). The asterisk marks a recording artifact (see Materials and Methods). Scale bars represent 300 µm except in (A), where it indicates 150 µm, and in (E and G), where it indicates 50 µm.

Since Krox20 is also expressed in r3, we next tried to determine which commissures could be affected at this level. Most auditory brainstem nuclei send axons across the midline, allowing binaural sound localization [21]. The projection from globular bushy cells in the anteroventral cochlear nucleus (aVCN), the first central auditory relay in the central nervous system, to the principal cells of the median nucleus of the trapezoid body (MNTB) is one of the most studied auditory commissures (Figure S6). We found that, at E13–E15, when aVCN axons first cross the floor plate [22], aVCN neurons expressed high-level *Robo3* mRNA (Figure 6A and 6B) and their axons expressed Robo3 protein (Figure 6C).

Genetic fate-mapping has shown that aVCN neurons are generated in the rhombic lip at the r2–r3 level [23]. We therefore studied aVCN-MNTB projections in *Krox20::cre;Robo3^lox/lox^* mice. As previously described [24], DiI injections in the aVCN of P12 control mice labeled aVCN axons projecting to the contralateral MNTB (*n* = 4/4; Figure 6D and 6E), where they form characteristic giant synapses called “calices of Held.” By contrast, in *Krox20::cre;Robo3^lox/lox^* mutants, this projection was exclusively ipsilateral, although typical calices of Held still formed on MNTB neurons (*n* = 4/4; Figure 6F and 6G). The numbers of labeled calices were not significantly different between controls and mutants (468±33 and 480±35 calices per side per animal, respectively, *p* = 0.7; *n* = 4 animals for each genotype; Student *t*-test with Mann-Whitney hypothesis). The aVCN ipsilateral projection to the superior olive appeared normal, and the olivocochlear bundle still crossed the midline (unpublished data). These results also incidentally show that globular bushy cells originate exclusively from r3 and that the *Robo3lox* mice can be used for genetic fate-mapping of hindbrain commissural neurons.

We next examined the consequence of the absence of a crossed aVCN/MNTB projection on auditory brainstem responses (ABR; see Materials and Methods) [25]. The detection thresholds of ABR did not significantly differ in *Krox20::cre;Robo3^lox/lox^* versus control mice and remained in the normal range from 5–40 kHz (Figure S6). In all animals, all waves were detected once the stimulus level was set above ABR threshold (Figure 6H). This indicates that neither auditory sensitivity nor gross neural conduction were affected. In control mice, the ABRs exhibited the four classical waves (I–IV) ipsilaterally (Figure 6H; for wave interpretation, see Materials and Methods and Text S1). Only two waves (IIIc and IVc) were recorded contralaterally, and their detection occurred with enough delay to be ascribed to contralateral excitation. The shape of mutant ABR waves was markedly different from controls. First, only three waves were present ipsilaterally (Figure 6H). At 60 dB sound pressure level (SPL), the latencies and amplitudes of waves I and II exhibited no significant difference relative to controls, which was not surprising, because the mutation targeted neural pathways beyond the cochlear nucleus. In contrast, in mutants, the next visible wave (III) had a longer latency (Figure 6H) than in controls. Moreover, in mutants, the IIIrd wave was not followed by any identifiable waves. Secondly, the contralateral wave IVc still occurred, but with a 0.42-ms average delay compared to the normal IVc. Its magnitude tended to be reduced although not significantly (*n* = 12 wild type versus *n* = 8 mutants; *p* = 0.06). In both mutants and controls, a similar dependency of wave latency on stimulus level was observed (unpublished data). These results show that in *Krox20::cre;Robo3^lox/lox^* mutants, the ipsilateral projection of globular bushy cells to MNTB neurons results in abnormal ABRs both ipsilaterally and contralaterally.

### An Impaired Interolivary Commissure Affects Locomotion

**Figure 7:**
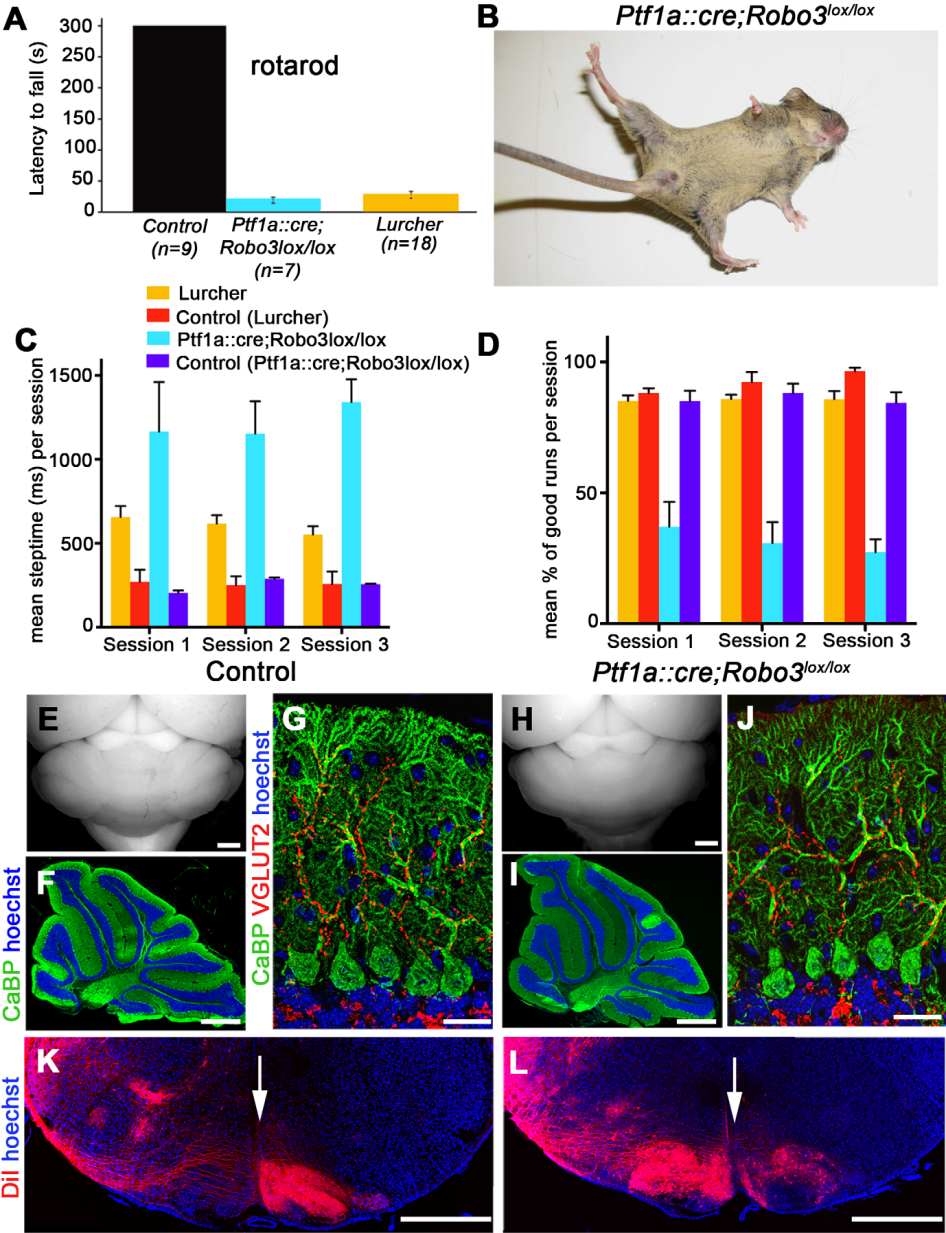
Uncrossed inferior olivary axons cause ataxic gait. (A) *Ptf1a::cre;Robo3^lox/lox^* mice are severely ataxic, which is demonstrated by a very short latency to fall in the Rotarod test (*n* = 7 mutants versus *n* = 9 for controls). (B) Image of a 4-mo-old *Ptf1a::cre;Robo3^lox/lox^* mouse displaying an ataxic gait. (C) The step time of *Ptf1a::cre;Robo3^lox/lox^* mice (light blue; *n* = 6) on the Erasmus Ladder is longer than in controls (violet and red; *n* = 4; *p*<0.001; multiple comparisons) or in *Lurcher* mice (orange; *p*<0.001; multiple comparisons). (D) *Ptf1a::cre;Robo3^lox/lox^* mice (light blue) also show significantly less successful trials per session than *Lurcher* mice (orange) (*p*<0.001 for all sessions; Mann-Whitney *U*-test, one-tailed). A trial was defined as successful if the mice were able to walk on the ladder without disruption (twisting, turning, walking backwards, etc.). (E to J) The morphology of the cerebellum in controls (E to G) and *Ptf1a::cre;Robo3^lox/lox^* mice (H to J) is comparable, and in both cases, their climbing fibers (the terminal arbors of inferior olivary axons) innervate the cerebellar cortex. (F and I) The size of the cerebellum and its foliation are similar as shown on sagittal sections of P32 control and *Ptf1a::cre;Robo3^lox/lox^* mice labeled with anti-calbindin antibodies and Hoechst. (G and J) VGLUT2-positive climbing fibers properly innervate CaBP+ Purkinje cell dendrites. (K and L) Coronal sections of P1 mice with unilateral injection of DiI in the cerebellum. The arrow marks the midline. In control (K), DiI-labeled inferior olivary (IO) neurons are exclusively found on the contralateral side, whereas in *Ptf1a::cre;Robo3^lox/lox^* mice (L), most IO neurons are traced on the ipsilateral side, and their axons do not cross the midline. Scale bars represent 500 µm except in (G and J), where they indicate 25 µm, and in (K and L), where they indicate 150 µm.

Inferior olivary (IO) neurons are the source of climbing fiber input on cerebellar Purkinje cells. During development, IO neurons migrate tangentially from the rhombic lip towards the floor plate. Upon reaching the floor plate, IO neuron cell bodies stop and only their axons cross it. We showed previously that IO axons are unable to cross the midline in *Robo3^−/−^* embryos and, as a result, project to the ipsilateral cerebellum [8]. However, the functional consequence of this ipsilateral rerouting of IO axons is unknown. To attempt to generate mice lacking the interolivary commissure, we crossed *Robo3^lox/lox^* conditional knockout with mice in which Cre recombinase was knocked into the *Ptf1a* (*Ptf1-p48*) locus (*Ptf1a::cre*) [26], which encodes a bHLH transcription factor expressed by a majority of IO neuron progenitors. The resulting *Ptf1a::cre;Robo3^lox/lox^* mice were viable, until at least 7 mo, but from postnatal day (P)10 onwards, they exhibited profound locomotor deficits (Figure 6A and 6B; see also Video S1) including an ataxic gait (35/35 cases) that persisted into adulthood. When locomotion was tested on the Rotarod, *Ptf1a::cre;Robo3^lox/lox^* mice showed a significant deficit in motor performance (Figure 7A and 7B); the *Ptf1a::cre;Robo3^lox/lox^* mice fell off the Rotarod at highly significant shorter latencies than controls (*p*<0.001, Mann-Whitney *U*-test, *n* = 7 mutants versus 9 controls). These motor performance deficits in the *Ptf1a::cre;Robo3^lox/lox^* mice were so severe that it appeared as if their ataxia was worse than that of mice that have no cerebellar output at all. We therefore compared their performance to that of *Lurcher* mice, which are characterized by a degeneration of their Purkinje cells and therefore by a virtual absence of the output from their cerebellar cortex [27],[28]. Although not significant (*p* = 0.2; Mann-Whitney *U*-test), the impaired locomotion in the *Lurcher* mice on the Rotarod was indeed milder than in the *Ptf1a::cre;Robo3^lox/lox^* mice (Figure 7A). On the more discriminative Erasmus Ladder [29], the difference in motor performance between *Ptf1a::cre;Robo3^lox/lox^* mice and *Lurcher* mice was fully apparent (Figure 7C and 7D); both the step time and the overall walking pattern (percentage of successful walking trials) were highly significantly more affected in *Ptf1a::cre;Robo3^lox/lox^* mice than in *Lurcher* mice (*Ptf1a::cre;Robo3^lox/lox^* versus *Lurcher* mice for step time in all sessions: *p*<0.001, multiple comparisons; for walking pattern in all sessions: *p*<0.001; Mann-Whitney *U*-test; see also Table S2). These findings indicate that the level of cerebellar ataxia induced by rewiring the climbing fiber projection is more severe than that caused by a functional loss of the cerebellar cortex, suggesting a pathological dominant-negative effect in the *Ptf1a::cre;Robo3^lox/lox^* mice. Despite this clear functional phenotype, the size and foliation of the cerebellum was unaffected in *Ptf1a::cre;Robo3^lox/lox^* mice (Figure 7E–7J; *n* = 3 mutants versus 3 controls). In addition, the distribution, morphology and immunostainings of their Purkinje cells, molecular layer interneurons, and granule cells (Figure S7) as well as the arborization of their climbing fibers (Figure 7G and 7J) were unaffected.

When DiI was injected unilaterally in the cerebellum of newborn controls, it retrogradely labeled IO neurons exclusively on the side opposite to the injection in control mice (4 out of 4 cases; Figure 7K). In contrast, following injections in *Ptf1a::cre;Robo3^lox/lox^* mice (8/8 cases; Figure 7L) about 67% of the DiI-labeled IO neurons were situated on the side of the injection, whereas 33% of the olivary axons projected contralaterally (Figure 7L). Immunolabeling of E13 *Ptf1a::cre;Robo3^lox/lox^* embryos (*n* = 4) with antibodies to Brn3.2, a marker of a large subset of IO neurons, showed that, as previously described in *Robo3* ^−/−^ mice, the ipsilateral IO projection did not result from a migration of IO cell bodies across the midline (Figure S8). The use of multiple IO neuron markers (Brn3.2, CaBP, BEN/SC1, CGRP) revealed that the lamellar organization of the IO was perturbed in *Ptf1a::cre;Robo3^lox/lox^* mice (*n* = 4; Figure S8), whereas the mossy fiber inputs and their neuronal sources developed normally (Figure S7).

**Figure 8:**
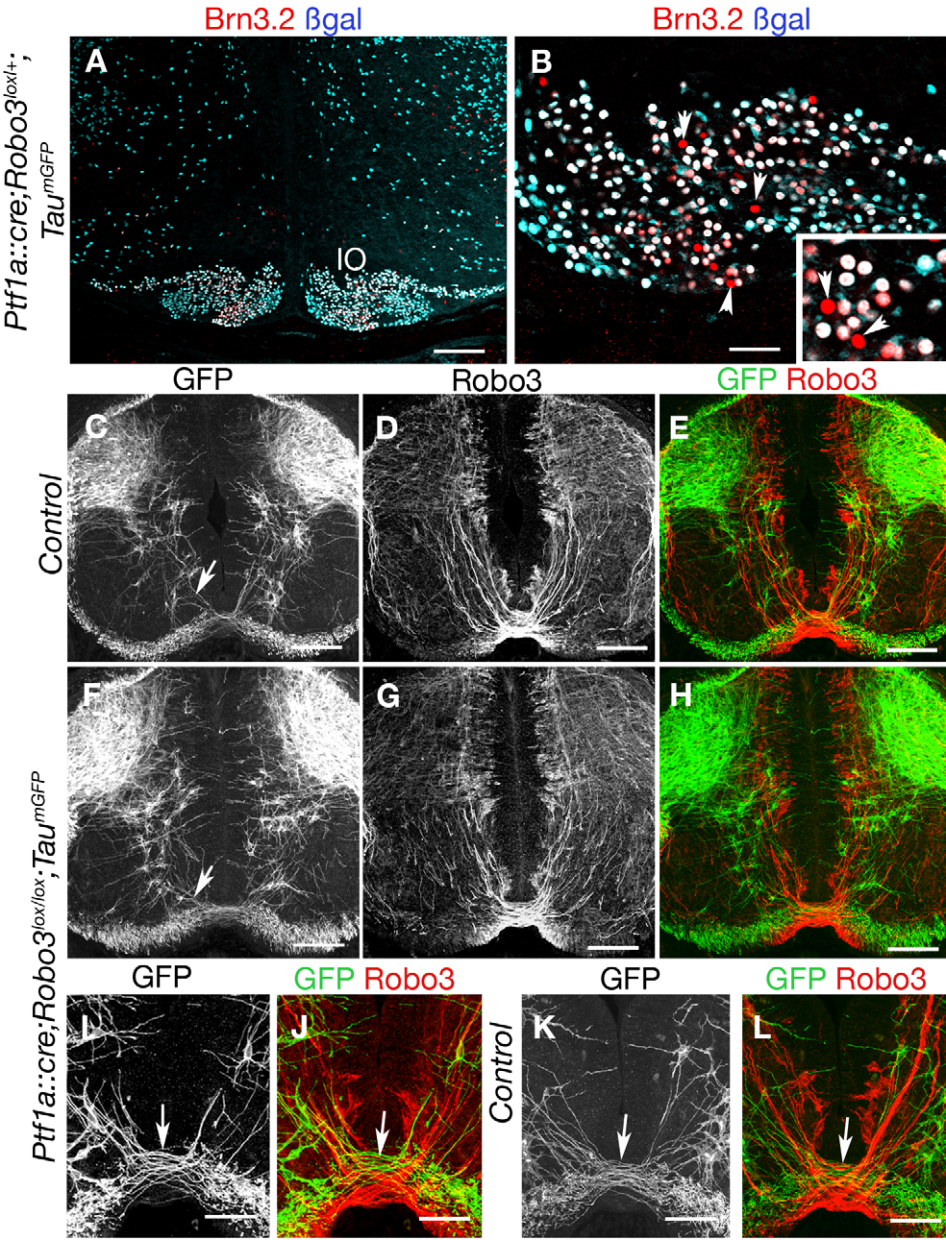
Analysis of *Ptf1a::cre;Robo3^lox/lox^;Tau^mGFP^* mice. (A and B) Coronal sections of the hindbrain at the level of inferior olive of E16 *Ptf1a::cre;Tau^mGFP^* embryo labeled with anti-βgal and Brn3.2 antibodies. Some Brn3.2-positive neurons in the inferior olive do not express the βgal (arrowheads and inset). (C–L) Coronal sections of the spinal cord at the level of the forelimbs in E13 *Ptf1a::cre;Robo3^lox/+^;Tau^mGFP^* (C–E, K, and L) *or Ptf1a::cre;Robo3^lox/lox^;Tau^mGFP^* (F–H, I, and J) embryos labeled with anti-GFP and anti-Robo3 antibodies. Most GFP-positive axons are in the dorsal spinal cord, and only a small subset of GFP-positive axons (short arrows) cross the floor plate in *Ptf1a::cre;Robo3^lox/+^;Tau^mGFP^* (C and K) but do not express Robo3 (E and L). This subset of GFP-positive commissural axons is still observed in *Ptf1a::cre;Robo3^lox/lox^;Tau^mGFP^* embryos (F and J). Scale bars represent 100 µm except in (B and I–L), where they indicate 50 µm.

To confirm that the ataxic behavior of *Ptf1a::cre;Robo3^lox/lox^* mice can be primarily attributed to the ipsilateral rerouting of a majority of olivocerebellar axons, we crossed these mice to a *Tau-lox-Stop-lox-mGFP-IRES-nls-lacZ* knock-in line [30] (here, called *Tau^mGFP^*). Upon Cre recombination in neurons, the Stop cassette is excised, leading to the permanent expression of a myristoylated GFP in axons and of β-galactosidase (βgal) in nuclei. This strategy made it possible to identify the neurons that expressed Cre recombinase in the *Robo3lox* background. In the inferior olive of *Ptf1a::cre;Robo3^lox/lox^* E16 embryos (*n* = 3), we found that 87±2% of Brn3.2-positive neurons also expressed βgal, whereas the remaining Brn3.2-positive neurons were completely negative for βgal (Figure 8A and 8B). This suggests that Cre is not expressed by all IO neurons in the *Ptf1a::cre* line, and could explain the maintenance of a contralateral contingent of IO axons in *Ptf1a::cre;Robo3^lox/lox^* animals (see Discussion). To find out whether Cre was expressed by spinal cord commissural neurons during midline crossing, we studied βgal and GFP expression in thoracic cross-sections of the spinal cord of E11–E13 *Tau^mGFP^* embryos at the level of the forelimbs (*n* = 3; Figure 8C–8L). We found that in the spinal cord of *Ptf1a::cre;Robo3^lox/+^;Tau^mGFP^* E11–E13 embryos, GFP-expressing axons were not immunoreactive for Robo3 and that commissures were not GFP-positive, with the exception of a very small ventral subset of axons (Figure 8). Interestingly, this small subset of GFP axons still crossed the midline in *Ptf1a::cre;Robo3^lox/lox^;Tau^mGFP^* embryos (Figure 8; *n* = 3). Moreover, Robo3-immunoreactive commissural neurons did not express βgal (*n* = 3 animals; unpublished data). The use of additional markers such as TAG-1 and neurofilament confirmed that spinal cord commissures were similar in control and *Ptf1a::cre;Robo3^lox/lox^* E13 embryos (Figure S9). As shown previously [31], in *Ptf1a::cre;Robo3^lox/+^;Tau^mGFP^* E12–E16 embryos, we could not detect βgal or GFP expression in the main sources of mossy fiber projection, the pontine neurons, the external cuneatus nucleus, or the lateral reticular nucleus (unpublished data).

## Discussion

We show that a *Robo3*-conditional allele can be used to genetically disrupt distinct commissural projections and force axons to project ipsilaterally. In all three systems studied here, commissure rewiring induces severe and permanent dysfunction of specific neuronal networks. Unlike surgical ablation of a given commissure, this strategy ends up rewiring commissural neurons on the ipsilateral side, but does not interrupt the activity of the targeted circuit. We show that for the systems studied here, ipsilaterally rerouted axons still connect to their proper targets. Moreover, the fact that human HGPPS patients do not exhibit major motor or cognitive impairments also suggests that the ipsilateral axons are fully functional. However, it is difficult to link the observed behavioral phenotypes exclusively to the loss of contralateral connectivity as there is also a rerouting in the targeting of these neurons that potentially could cause novel and perturbed neuronal signals leading to a defect, which is not caused by the lack of commissural connections per se but rather by the new wiring.

Our results and others [8],[9] suggest that Robo3 expression is required for most, if not all, commissural axons to cross the floor plate throughout spinal cord and hindbrain. The persistence of commissural axons that continue to cross the floor plate in domains expected to express cre recombinase in the *Robo3* conditional lines presented here may be explained in a number of ways. In *Krox20::cre;Robo3^lox/lox^* embryos, the few axons crossing at r3 and r5 level may originate from neurons located in adjacent rhombomeres as it is well known that many commissural axons extend rostrally and caudally before crossing [17],[32], and that during development, mixing of neurons from adjacent rhombomeres does occur [33],[34] (one example is provided by facial branchiomotor neurons that migrate from r4 to r6 [35]). Another reason for incomplete rerouting of commissural axons is that Cre recombinase may not be expressed by all r3/r5 neurons, as is the case for IO neurons in *Ptf1a::cre;Robo3^lox/lox^* knockout. Finally, cre might be expressed too late, after these commissural axons have crossed, an hypothesis we favor to explain the persistence of the ventral subset of commissural axons in the spinal cord of *Ptf1a::cre;Robo3^lox/lox^* embryos.

Although Robo3 is required for commissures to form in the hindbrain and spinal cord, this receptor is not sufficient for crossing as it is also expressed by many noncommissural neurons in the forebrain and hindbrain [36], such as the abducens motor neurons that project ipsilaterally. This opposite behavior of some Robo3-expressing axons could be attributed to a differential expression in commissural/noncommissural axons of Robo3 partners, possibly Robo1 and Robo2, or of a different Robo3 isoform. A recent study has shown that two Robo3 splice variants that differ in their cytoplasmic tail have distinct activities (pro-crossing and anti-crossing) in axons [37].

We found that deletion of *Robo3* selectively in two hindbrain rhombomeres, including that comprising the abducens nucleus, yields mice that display selective horizontal eye movement defects reminiscent of those in the human HGPPS patients [12]–[15]. Likewise, zebrafish *Robo3* mutants exhibit oculomotor defect [38]. Since there is, to the best of our knowledge, no oculomotor center originating from r3, our results in mice indicate that commissural projections from r5, most likely internuclear oculomotor connections, serve to facilitate large amplitude, compensatory eye movements in the horizontal plane. Our results also suggest that the absence of this commissure is one of the primary causes of the eye movement defects in HGPPS patients, who also suffer from a mutation in the *Robo3* gene [11],[12]. Accordingly, abducens motor axons project normally in *Krox20::cre;Robo3^Iox/lox^* mice. Moreover, we found that horizontal eye movements are not perturbed in *Islet1::cre;Robo3^lox/lox^* mice, which lack Robo3 in abducens motor neurons (unpublished data). These results do not exclude the possibility that oculomotor defects in HGPPS patients may also involve other supranuclear centers such as the pons and cerebellum, which could henceforth be studied in transgenic mice by deleting Robo3 in appropriate regions using the conditional allele.

We show that in mice, in the absence of Robo3, the aVCN-MNTB projection is entirely uncrossed and that this results in strong defects in ABR. The most striking feature of ABRs in *Krox20::cre;Robo3^lox/lox^* mice is the presence on the ipsilateral side of a much-delayed wave III not followed by any later wave. This abnormal wave III could involve wrongly rewired neurons, and its long delay suggests that the corresponding pathway does not function in a strictly normal manner. Contralaterally, the persistence of waves identifiable as IIIc and IVc may be attributed to crossing pathways originating outside of r3–r5. Although there are species differences in the organization of auditory circuits [39],[40], our results suggest that the trapezoid body could be uncrossed in HGPPS patients, at least partially. It will be of interest to determine whether sound localization is perturbed in HGPPS patients, some of whom also seem to have abnormal ABR [13],[14].

Interestingly, HGPPS patients have a hypoplastic pons, an uncrossed corticospinal tract [12],[13], and most likely uncrossed spinal cord and interolivary commissures. Yet these patients are still coordinated and not ataxic. This contrasts with the severe ataxic gait of *Ptf1a::cre;Robo3^lox/lox^* mice. In this mouse line, spinal cord commissures and the corticospinal tract (unpublished data) do not seem to be affected, and among embryonic precerebellar neurons, cre recombinase is only expressed by IO neurons and not by mossy fiber projection neurons. Possibly, a selective reduction of the interolivary commissure results in a more dramatic locomotion impairment than a nonselective rewiring of multiple parts of the motor system in which, presumably, the different parts of the nervous system are ultimately still connected to the correct sides of the other relevant parts. Moreover, the selective reduction of the interolivary commissure in the *Ptf1a::cre;Robo3^lox/lox^* mice also results in a stronger locomotion impairment than mice without any cerebellar output (i.e., the *Lurcher* mice). This phenotype of the *Ptf1a::cre;Robo3^lox/lox^* mice on the Erasmus Ladder can be explained by the fact that the olivocerebellar system may operate in a push–pull fashion [41], because converting the “pull” into a “push” by reversing the modulation of climbing fiber activities should indeed result in a phenotype that is more severe than having no output of the cerebellar cortex at all. Thus, taken together, our results suggest that having only one component of a commissural circuit misrouted and uncrossed may be functionally worse than having all of its components affected or having no output at all.

Many studies have shown that upon entering and crossing the floor plate, commissural axons receive signals that induce major and long-term modification of their behavior and prevent them from re-entering the midline. This is associated with a loss of attraction for netrin-1 [42] involving DCC silencing and DCC/Robo dimerization [43], and an activation of Slit- and Semaphorin-mediated repulsion [44]. Moreover, there is also evidence that the translation of some axon guidance receptors [45] or their splicing is modulated by floor plate crossing [37]. However, it was not known if all these molecular changes were required for crossed axons to recognize and follow their correct pathway on the contralateral side and, more importantly, to reach their final target cells. Our results suggest that even in the absence of midline crossing, both olivocerebellar axons and aVCN axons still contact their appropriate target neurons (Purkinje cells and MNTB, respectively), but on the ipsilateral rather than on the contralateral side. These findings raise the possibility that midline crossing in general may not have a major function in axon guidance after crossing, and that its major importance is only to allow, at a physiological level, the integration of inputs coming from both sides of the nervous system. Future studies will have to confirm this at other axial levels and in other phyla like Protostomia.

## Materials and Methods

### Mouse Lines

C57BL/6 mice (Janvier) were used for expression studies. Mice were anesthetized with isofluorane (Abbott). The day of vaginal plug is embryonic day 0 (E0), and the day of birth corresponds to postnatal day 0 (P0).

The *Robo3* conditional knockout mouse line was established at the MCI/ICS (Mouse Clinical Institute–Institut Clinique de la Souris, Illkirch, France; http://www-mci.u-strasbg.fr). To generate the *Robo3* conditional knockout, the targeting vector was constructed as follows. Three fragments of 5.1, 1.1, and 3.7 kb (respectively, the 5′, floxed, and 3′ arms) were amplified by PCR using 129S2/SvPas DNA as template and sequentially subcloned in an MCI proprietary vector. This MCI vector has a floxed neomycin resistance cassette. The linearized construct was electroporated in 129S2/SvPas mouse embryonic stem (ES) cells. After selection, targeted clones were identified by PCR using external primers and further confirmed by Southern blot with 5′ and 3′ external probes. Two positive ES clones were injected into C57BL/6J blastocysts, and derived male chimaeras gave germline transmission. The *Robo3*-null knockout line was described previously [9]. Briefly, it consists on a knock-in of the *GFP* gene into the first exon of *Robo3*, leading to the expression of the GFP by Robo3-expressing cells. However, the GFP signal is too weak to be seen without the use of an anti-GFP antibody. The *Hb9::GFP* mice have a transgene containing a 9-kb-long region of the Hb9 promoter that drives eGFP expression in all postmitotic somatic motor neurons [18]. The GFP signal is bright enough to be seen directly. The *Ptf1a::cre* and *Krox20::cre* knock-in lines were previously described [16],[26]. Homozygous *Krox20^cre/cre^* are embryonic lethal and were therefore maintained as heterozygous for Cre. The *Tau^mGFP^* knock-in line was obtained by replacing the coding sequence of the Tau gene by a *lox-Stop-lox-mGFP-IRES-nls-lacZ* cassette [30]. Unless otherwise mentioned, controls were *Robo3^+/−^* or *Robo3^lox/lox^* animals or double heterozygotes that were always found to be undistinguishable from wild-type mice. All mice were genotyped by PCR (see Table S3 for primer sequences). All animal procedures were carried out in accordance with institutional guidelines (UPMC and INSERM).

### Histology and Immunocytochemistry

Embryos and mice were processed as described previously [46]. Tissue sections and whole-mount embryos were hybridized with digoxygenin-labeled riboprobes as described previously [46].

The following primary antibodies were used: mouse anti-CaBP (1∶2,000, Swant), mouse anti-neurofilament (1∶1,000, gift from Virginia M.-Y. Lee, Philadelphia, PA), mouse anti-Islet1 (1∶100, Developmental Studies Hybridoma Bank, University of Iowa), mouse anti-parvalbumin (1∶1,000, Swant), mouse anti-Robo3 (1∶200, R&D which recognizes the N-terminal domain of Robo3), guinea pig anti-VGLUT2 (1∶1,000, Millipore), rabbit anti-Hb9 (1∶1,000, Abcam), rabbit anti-CGRP (1∶1,000, Peninsula), rabbit anti-GFP (1∶300, Invitrogen), rabbit anti-Tag1 (1∶3,000, gift from Dr. Domna Karagogeos, University of Crete Medical School, Heraklion, Greece), rabbit anti-CaBP (1∶5,000, Swant), rabbit anti-βGal (1∶1,000, Cappel), goat anti-mouse Alcam (1∶200, R&D), goat anti-Brn3.2 (1∶200, Santa Cruz Biotechnology), goat anti-Robo3 (1∶300, R&D), goat anti-ChAT (1∶400, Millipore), and chicken anti-GFP (1∶800, Abcam). The following secondary antibodies were used: donkey anti-goat, anti-mouse, anti-rabbit, and anti-guinea pig coupled to CY3 or CY5 (1∶600, Jackson Laboratories), donkey anti-goat, anti-mouse, anti-rabbit and anti-chicken coupled to Alexa Fluor 488 (1∶600, Invitrogen). Sections counterstained with Hoechst 33258 (10 µg ml^−**1**^, Sigma) were examined with a fluorescent microscope (DM6000, Leica) coupled to a CoolSnapHQ camera (Roper Scientific) or a confocal microscope (FV1000, Olympus).

### Quantifications

The number of abducens motor neurons was quantified on 20-µm coronal sections of adult hindbrain double labeled with anti-ChAT and anti-Hb9. Neurons were counted on alternating sections and counts multiplied by two to better reflect the total number of neurons in each abducens nucleus.

Di-labeled IO neurons were counted as follows: on every section containing the inferior olive, four confocal optic planes were acquired (two on the contralateral side and two on the ipsilateral side), and only the cells showing circle-shaped DiI labeling were counted as positive (see Figure S8). The percentage of ipsilateral and contralateral neurons represents the number of ipsilateral- or contralateral-labeled neurons divided by the total number of traced neurons on both sides.

The percentage of (βgal−; Brn3.2+) olivary neurons was calculated as the ratio of (βgal−; Brn3.2+) on (βgal+; Brn3.2+) neurons. Immunolabeled nuclei were counted on 20-µm sections of E16 inferior olives (one section out of five was considered from three embryos).

The number of DiI-labeled calyces of Held were counted from stacked confocal planes (step size: 1.16 µm) encompassing 50 µm of 100-µm vibratome sections, on all the sections containing the MNTB. Only typical “moon crescent”–shaped objects were counted as calyces (see Figure 6E and 6G). Quantification in the text are given as the number of calyces per nuclei (only one side) per animal. This number is an underestimate of the real number of calyces, because the confocal stack does not encompass the entire section.

### In Situ Hybridization

Antisense riboprobes were labeled with digoxigenin-11-D-UTP (Roche Diagnostics) as described previously [46] by in vitro transcription of cDNAs encoding *Robo3* [8], *Barhl1* [47], *Slit1–Slit3* [46], *Netrin1* [48], and *Shh* [49]. A mouse *Robo3* cDNA specific for exons 12–14 was amplified by PCR with the following primers: 5′-CGGAATTCTGGTATTCAGTGATGACCCC-3′ and 5′-GCTCTAGAACAGCAGCCTATCTAGGCCA-3′ and cloned into pBluescript vector. The anti-sense probe was synthesized in vitro by digesting the construct with XbaI and using T7 RNA polymerase. Control sense probe yielded no signal.

### DiI Tracing

The 4% PFA–fixed embryos or postnatal animals were injected with small crystals of DiI (Molecular Probes) using glass micropipettes. Injected brains were kept at 37°C for 1 wk (aVCN-MNTB and VI-III commissures) to 3 wk (olivocerebellar projection). Brains were cut in 100-µm sections with a vibratome (Leica) and counterstained with Hoechst.

### Eye Movement Recordings

Mice were placed in a restrainer, fixed onto the center of a turntable, facing a cylindrical screen, and their OKR and VOR measured as previously described [19]. See Text S1 for a detailed description of this procedure.

### Rotarod Training

Mice were placed on the cylinder of a Rotarod apparatus (model 7650, Ugo Basile Biological Research Apparatus) that rotated at four turns per minute, and the time the mice spent on top of the cylinder was recorded. After 300 s, the recording was ended. The animals were tested twice, and between the sessions, there was a 60-min break during which the mice rested in their cages. Mean values of the two trials were calculated for each animal. The Mann-Whitney test was used for statistical analyses (*p*<0.05 was considered significant).

### Erasmus Ladder

The Erasmus ladder [29] is a fully automated system designed to screen motor performance and motor learning. It consists of a horizontal ladder that is composed of 2×37 rungs (pressure sensors) and is situated between two sheltered boxes equipped with pressurized air outlets. In order to test motor performance *Ptf1a::cre;Robo3^lox/lox^* mice (*n* = 6) and their littermates (*n* = 4) were placed in one of the shelters. After a randomized delay of 9–12 s, the light was turned on in the box, automatically followed after 3 s by an air puff from the pressurized air outlet, encouraging the mice to leave the shelter and walk across the ladder to the shelter on the other side, where the procedure was repeated. The paw placement and overall step time (the time needed to transfer the paw from one sensor to the other) was recorded for each trial in real time using the pressure sensors. One trial was defined as a crossing from one shelter to another. A trial was regarded successful if the mice walked with a consistent pattern, touching the pressure sensors with all paws, and with no disruptions such as rearing, twisting, or turning around. One session consisted of at least 20 trails.

### Recordings of Auditory Brainstem-Evoked Responses

Mice were anesthetized with a mixture of ketamine and levomepromazine. ABRs were collected between subcutaneous needle electrodes inserted at the vertex and mastoids on both sides, with the ground electrode in the back (see Text S1). Sound stimuli were short broadband clicks and frequency-specific tone-bursts (two-period rise–fall, 20-period plateau, frequency 5, 10, 15, 20, 32, and 40 kHz) presented at a repetition rate of 17 per second, the sound level varying in 5-dB steps from 10 to 90 dB SPL (with 0 dB SPL = 2.10^−5^ N.m^−2^). The responses from the electrodes were amplified (×100,000), filtered (10–3,000 Hz), digitally converted and averaged (×500) by a computerized data-acquisition system. On the ipsilateral side, wave I reflects the activity of spiral ganglion cells. Later waves II, III, and IV are ascribed to the sequential activation of more and more central neural generators. On the contralateral side, a short latency wave was observed similar to ipsilateral wave I (Figure 6H), which precluded its being due to the activation of neural pathways contralateral to the stimulus and was thus likely due to remote detection of responses from the ipsilateral spiral ganglion through electrically conductive neck tissues. The total absence of any deflection in the contralateral ABRs at the latency corresponding to ipsilateral wave II indicates that the artifact affecting this short latency contralateral wave did not come into play for later waves.

### Statistical Analysis

Unless mentioned in the text, results are presented as means±standard deviation (sd). All differences of the means between two sample sets were assessed by two-tailed Student *t*-test with Welsh hypothesis (unequal variances) or a Mann-Whitney *U*-test, as appropriate, except for the eye movement results. There curves were first tested with an ANOVA for repeated measurements for significant differences, and only for the different curves a Student *t*-test was used to identify significantly different points. Statistics were carried out with R software (http://www.r-project.org/).

## Supporting Information

Figure S1 **Schematic representation of the oculomotor system involved in lateral eye movement.** (A) In controls, lateral eye movements are controlled by two pairs of cranial motor nuclei, the abducens (VI, green) and the oculomotor (III, red), projecting ipsilaterally to the lateral rectus muscle (LR) and medial rectus muscle (MR) respectively. Conjugate eye movement involves a commissural connection (arrow) between VI nucleus interneurons (black) and nucleus III. (B) In *Robo3*-null embryos and *Krox20::cre;Robo3^lox/lox^* mutants, the III and VI still project to the correct muscle, but the internuclear commissure is severely reduced. It is still unknown whether VI interneurons innervate the ipsilateral nucleus III.
(0.30 MB TIF)

Figure S2 **Generation of a Robo3 conditional allele.** (A) To generate the Robo3 conditional allele, loxP sites were inserted around exons 12, 13, and 14 of the *Robo3* gene. Cre excision of exons 12–14 generates a Robo3 protein interrupted at the beginning of the second fibronectin type III repeat. This truncated Robo3 protein, which lacks the transmembrane and cytoplasmic domains, is unable to act as a receptor. (B) Sequence of the conditional *Robo3* allele around the loxP sites in the targeting vector. The exons are in red, the primers used for genotyping in green, the loxP sites in blue, and the Frt sites in violet.
(1.88 MB TIF)

Figure S3 **Expression pattern of midline-derived axon guidance factors.** Coronal sections at the level of rhombomere 5 of E11 embryos hybridized with riboprobes for *Netrin1* (A and C), *Shh* (C and D), *Slit1* (E and F), *Slit2* (G and H), and *Slit3* (I and J). The expression pattern is similar in controls and *Krox20::cre;Robo3^lox/lox^* embryos. Controls are either *Robo3^lox/lox^* (*Netrin1*, *Shh*, *Slit1*) or *krox20::cre;Robo3^lox/+^* (*Slit2*, *Slit3*). Scale bars represent 50 µm.
(3.82 MB TIF)

Figure S4 **Normal expression of motoneuron markers.** Coronal sections at the level of the abducens nuclei of adult (A–B′) and E13 (C–D) control (A, A′, and C) and *Krox20::cre;Robo3^lox/lox^* (B, B′, and D) animals. (A–B′) In both cases, abducens motoneurons are immunoreactive for ChAT (A and B) and CGRP (A′ and B′). (C and D) They also express islet1 (arrowheads). Note the abnormal shape and position of the abducens nuclei in *Krox20::cre;Robo3^lox/lox^* mutants. Scale bars represent 50 µm, except in (C and D), where they indicate 70 µm.
(1.08 MB TIF)

Figure S5 **Reduced internuclear commissure in Robo3-deficient mice.** (A–F) Coronal section of P0 brains immunostained with Hb9 following DiI tracing of the VI–III internuclear connection. In controls (A and B), the internuclear commissure is strongly labeled (arrow), and DiI-labeled cells are observed at the level of the abducens nucleus (VI). (C and D) In *Krox20::cre;Robo3^lox/lox^* mutants, the DiI-labeled internuclear commissure is severely reduced. Some DiI-labeled fibers are still found on the contralateral side, but no cells are traced in the vicinity of the abducens nucleus. In *Robo3^−/−^* knockout (E and F), the commissure is almost completely absent. No cells are traced on the contralateral side. Scale bars represent 100 µm.
(1.87 MB TIF)

Figure S6 **ABR thresholds and binaural difference in control and** ***Krox20::cre;Robo3^lox/lox^*** **mice.** (A) The detection thresholds of ABR did not significantly differ in *Krox20::cre;Robo3^lox/lox^* versus control mice and remained in the normal range from 5–40 kHz. (B) The binaural-difference wave complex resulting from the fact that the late ABR waves in response to a diotic stimulus are smaller than the sum of waves in response to stimuli in either the right or the left side (see Materials and Methods), was still present in mutants, with similar amplitudes and latencies. It suggests that at least part of the functional coupling of left and right signals remained present in *Krox20::cre;Robo3^lox/lox^* mice, likely in relation to commissural neurons outside r3 and r5. (C and D) Schematic representation of the auditory pathway in control (C) and *Krox20::cre;Robo3^lox/lox^* mice (D). (G) Auditory inputs (red arrowheads) from the hair cells are transmitted to neurons of the spiral ganglion that project ipsilaterally into the brainstem on globular bushy cells in the anterior part of the ventral cochlear nucleus (aVCN). In controls, these cells send large-diameter axons to the contralateral medial nucleus of the trapezoid body (MNTB), forming calyces of Held synapses (arrow). MNTB neurons then project to the lateral superior olive (SO). In *Krox20::cre;Robo3^lox/lox^* mice, globular bushy cell axons only project to the ipsilateral MNTB but still form calyces. (C) is adapted from [4]
(0.63 MB TIF)

Figure S7 **Normal cerebellar cortex and pontine nuclei in** ***Ptf1a::cre;Robo3^lox/lox^*** **mice** (A–D) Sagittal sections of the cerebellar cortex of P32 control (A and C) and *Ptf1a::cre;Robo3^lox/lox^* mice (B and D) labeled with antibodies against parvalbumin (Parv) and calbindin (CaBP) and counterstained with Hoechst. Purkinje cells coexpress the two proteins, whereas molecular layer interneurons only express parvalbumin. (C and D) were obtained by subtraction of the calbindin channel (green) from the parvalbumin channel (red). The morphology of Purkinje cells and the density of molecular layer interneurons are similar. (E and F) show a ventral view of whole-mount hindbrain of E15 embryos hybridized with *Barhl1* riboprobe. The stream of migrating pontine neurons (arrowheads) is comparable. Scale bars represent 25 µm, except in (E and F), where they indicate 500 µm
(2.25 MB TIF)

Figure S8 **Phenotype of inferior olivary neurons in** ***Ptf1a::cre;Robo3^lox/lox^*** **mice.** (A–F) Coronal sections of P0 (A, B, D, and E) and E13.5 (C and F) hindbrain at the level of the inferior olive labeled with Brn3.2 (A–F) and calbindin (A and D). The structure of the inferior olivary nucleus is disorganized in *Ptf1a::cre;Robo3^lox/lox^* mice (compare [A and B] with [D and E]), and many of its subdivisions have an abnormal shape. The arrows in (D) show the position of dorsal cap of Kooy (DC in [A]) and the β-nucleus (β in [A]) neurons, and the arrows in (E) indicate the disorganized principal olive (PO in [B]). (C and F) Brn3.2+ IO neurons do not cross the midline (arrow) in either control (C) or *Ptf1a::cre;Robo3^lox/lox^* (F) embryos. The arrowheads point to migrating LRN neurons. (G) is a 1.16-µm-thick confocal image of DiI-labeled IO neurons in control P0 mouse with Hoechst counterstaining. (H and I) Schematic representation of the olivocerebellar projection in control (H) and *Ptf1a::cre;Robo3^lox/lox^* mice (I). In control, all IO neurons project across the ventral midline to the contralateral cerebellum (Cer) where their terminal arborization, the climbing fibers (CF), synapse on Purkinje cells. In *Ptf1a::cre;Robo3^lox/lox^* mice, most IO axons project into the ipsilateral cerebellar cortex. Scale bars represent 100 µm, except in (G), where it indicates 20 µm a, b, and c indicate the subnuclei a, b, and c, respectively, of the MAO. DAO, dorsal accessory olive; DC, Dorsal Cap of Kooy; MAO, medial accessory olive.
(3.31 MB DOC)

Figure S9 **Normal spinal cord commissures in** ***Ptf1a::cre;Robo3^lox/lox^*** **embryos.** (A–H) are coronal sections of the spinal cord of E13 embryos immunolabeled with neurofilament and Robo3 (A, B, E, and F) or TAG-1 and Robo3 (C, D, G, and H). Commissures are not reduced and still express Robo3 in *Ptf1a::cre;Robo3^lox/lox^* mice. Scale bars represent 200 µm.
(2.74 MB TIF)

Table S1 (0.44 MB PDF)

Table S2 (1.59 MB PDF)

Table S3 (0.02 MB DOC)

Text S1 **Supplemental Methods.** Eye movement and ABR recordings.
(0.02 MB DOC)

Video S1 **Ataxic gait of** ***Ptf1a::cre;Robo3^lox/lox^*** **mice.** Two P15 littermates: the *Ptf1a::cre;Robo3^lox/+^* mouse walks normally, whereas the *Ptf1a::cre;Robo3^lox/lox^* mouse has an ataxic gait.
(1.36 MB MOV)

## Abbreviations

ABR: auditory brainstem response
aVCN: anterior vestibular cochlear nucleus
E: embryonic day
HGPPS: horizontal gaze palsy with progressive scoliosis
IO: inferior olivary
MNTB: median nucleus of the trapezoid body
OKR: optokinetic reflex
r: rhombomere
VOR: vestibulo-ocular reflex
VVOR: visual vestibulo-ocular reflex
