# Supplementary figures and images for: Genetic Dissection of the Function of Hindbrain Axonal Commissures
Source: PLoS Biol. 2010 Mar 9;8(3):e1000325. doi: 10.1371/journal.pbio.1000325 (PMC2834709; doi:10.1371/journal.pbio.1000325)

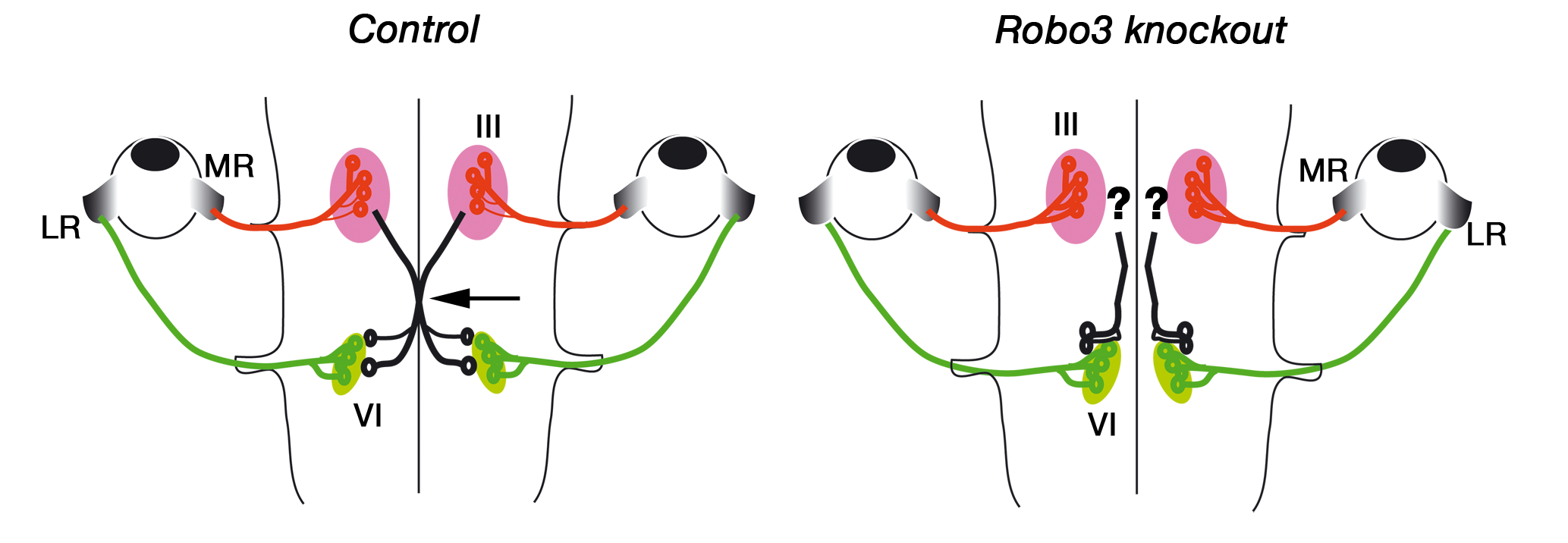

Supplement: Figure S1 — Schematic representation of the oculomotor system involved in lateral eye movement. (A) In controls, lateral eye movements are controlled by two pairs of cranial motor nuclei, the abducens (VI, green) and the oculomotor (III, red), projecting ipsilaterally to the lateral rectus muscle (LR) and medial rectus muscle (MR) respectively. Conjugate eye movement involves a commissural connection (arrow) between VI nucleus interneurons (black) and nucleus III. (B) In Robo3-null embryos and Krox20::cre;Robo3lox/lox mutants, the III and VI still project to the correct muscle, but the internuclear commissure is severely reduced. It is still unknown whether VI interneurons innervate the ipsilateral nucleus III. (0.30 MB TIF) [file pbio.1000325.s001.tif]

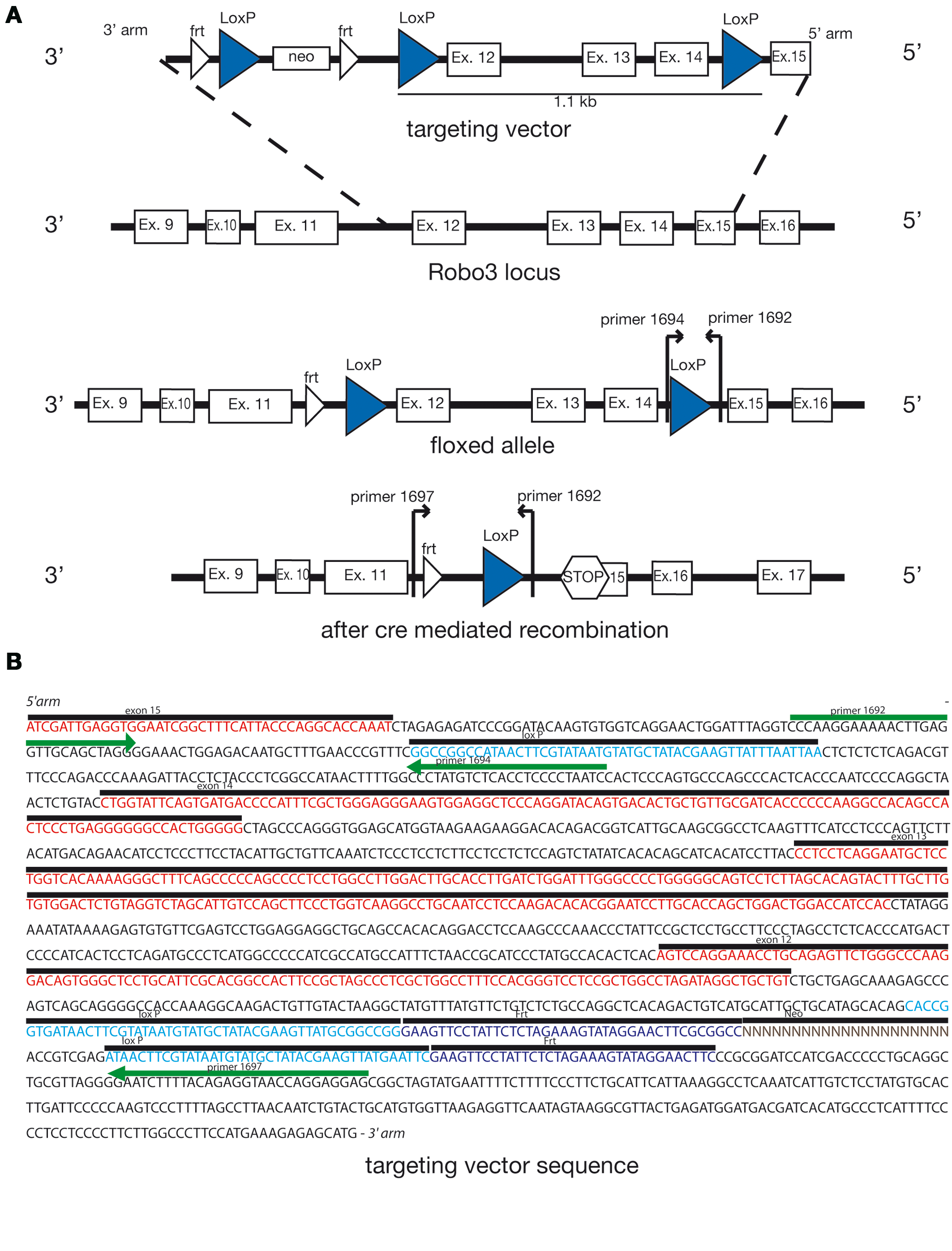

Supplement: Figure S2 — Generation of a Robo3 conditional allele. (A) To generate the Robo3 conditional allele, loxP sites were inserted around exons 12, 13, and 14 of the Robo3 gene. Cre excision of exons 12–14 generates a Robo3 protein interrupted at the beginning of the second fibronectin type III repeat. This truncated Robo3 protein, which lacks the transmembrane and cytoplasmic domains, is unable to act as a receptor. (B) Sequence of the conditional Robo3 allele around the loxP sites in the targeting vector. The exons are in red, the primers used for genotyping in green, the loxP sites in blue, and the Frt sites in violet. (1.88 MB TIF) [file pbio.1000325.s002.tif]

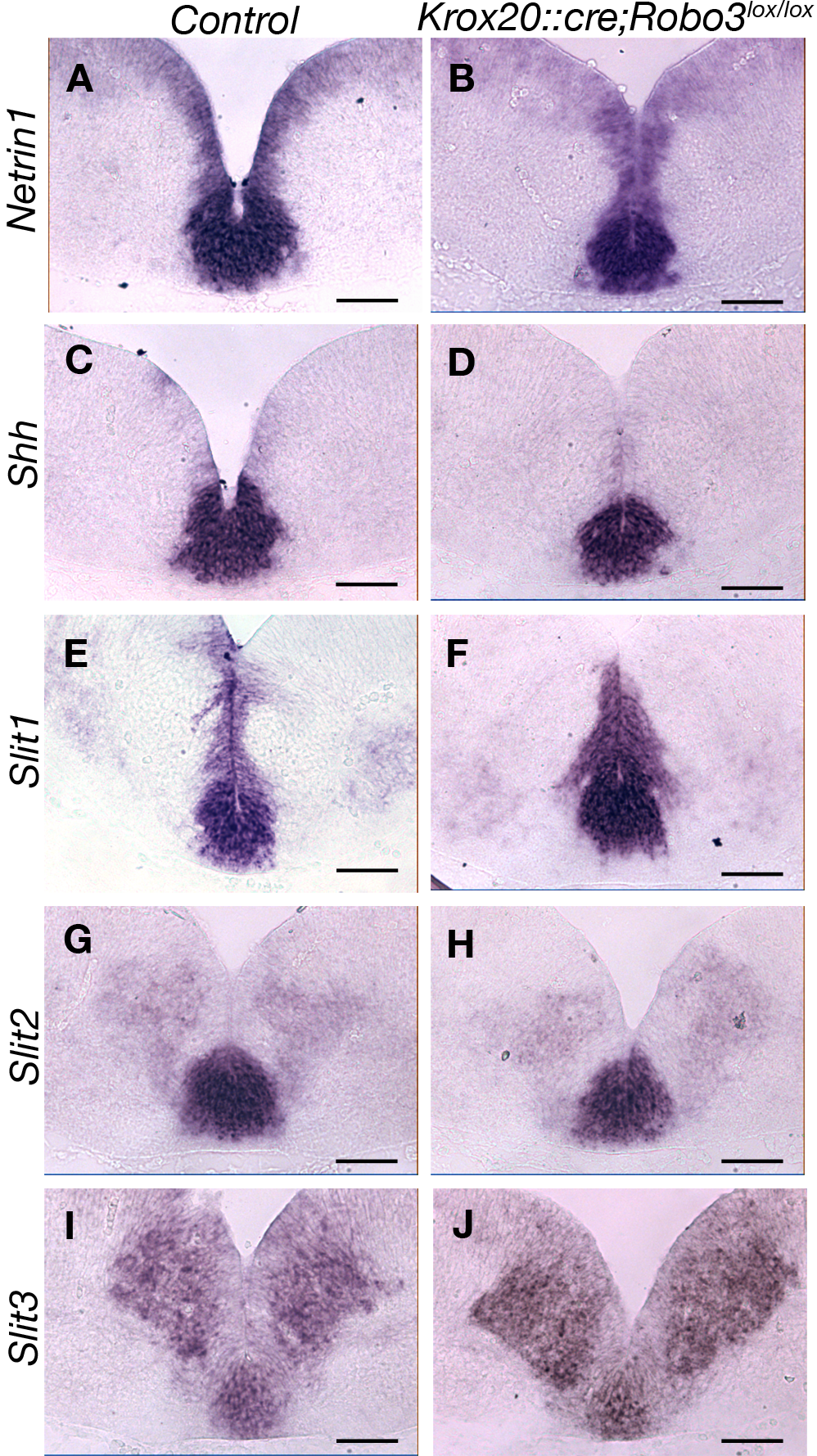

Supplement: Figure S3 — Expression pattern of midline-derived axon guidance factors. Coronal sections at the level of rhombomere 5 of E11 embryos hybridized with riboprobes for Netrin1 (A and C), Shh (C and D), Slit1 (E and F), Slit2 (G and H), and Slit3 (I and J). The expression pattern is similar in controls and Krox20::cre;Robo3lox/lox embryos. Controls are either Robo3lox/lox (Netrin1, Shh, Slit1) or krox20::cre;Robo3lox/+ (Slit2, Slit3). Scale bars represent 50 µm. (3.82 MB TIF) [file pbio.1000325.s003.tif]

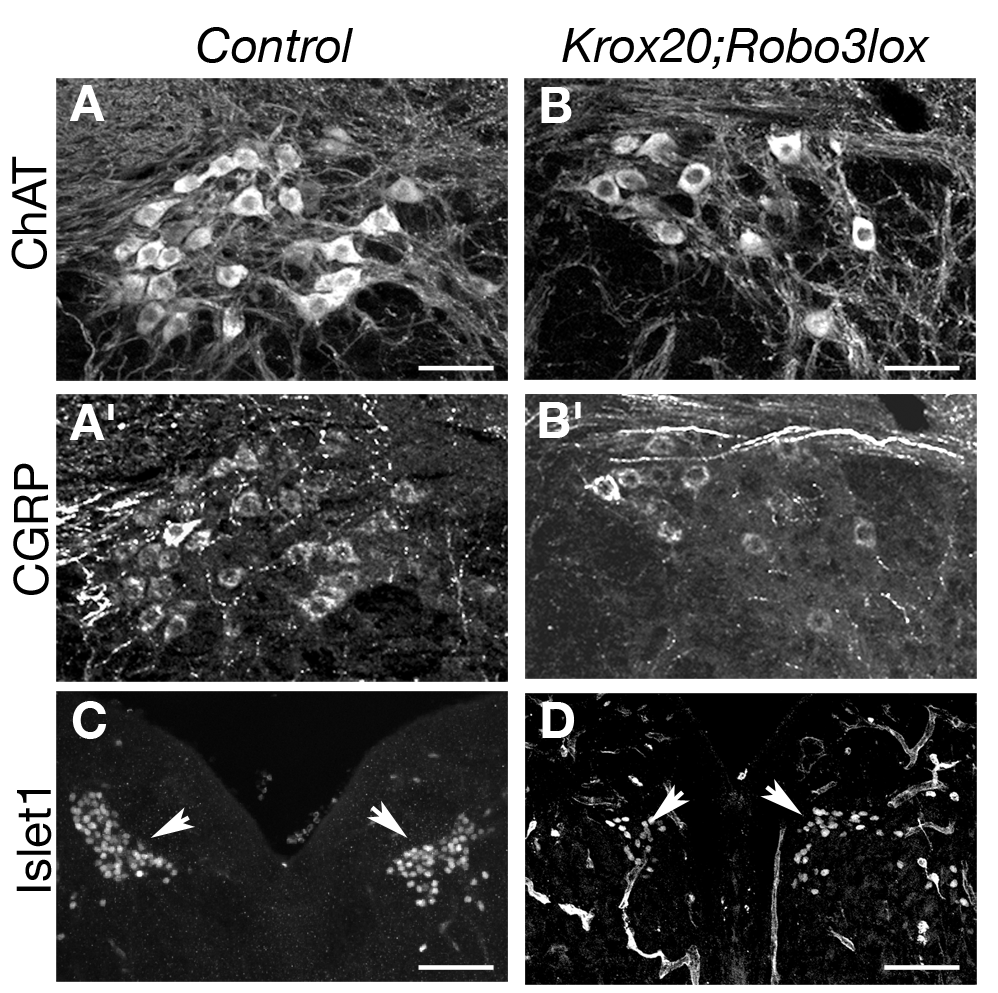

Supplement: Figure S4 — Normal expression of motoneuron markers. Coronal sections at the level of the abducens nuclei of adult (A–B′) and E13 (C–D) control (A, A′, and C) and Krox20::cre;Robo3lox/lox (B, B′, and D) animals. (A–B′) In both cases, abducens motoneurons are immunoreactive for ChAT (A and B) and CGRP (A′ and B′). (C and D) They also express islet1 (arrowheads). Note the abnormal shape and position of the abducens nuclei in Krox20::cre;Robo3lox/lox mutants. Scale bars represent 50 µm, except in (C and D), where they indicate 70 µm. (1.08 MB TIF) [file pbio.1000325.s004.tif]

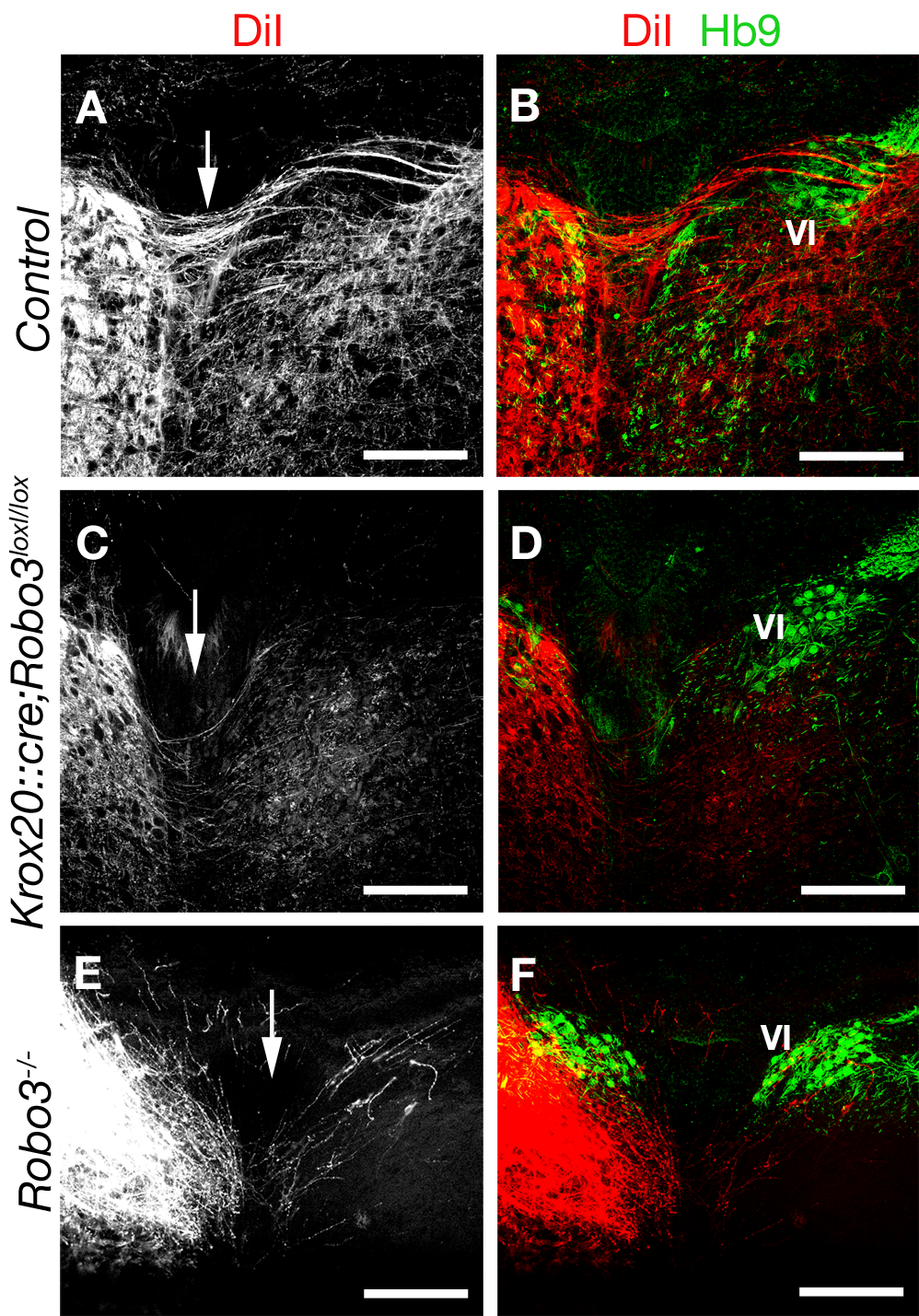

Supplement: Figure S5 — Reduced internuclear commissure in Robo3-deficient mice. (A–F) Coronal section of P0 brains immunostained with Hb9 following DiI tracing of the VI–III internuclear connection. In controls (A and B), the internuclear commissure is strongly labeled (arrow), and DiI-labeled cells are observed at the level of the abducens nucleus (VI). (C and D) In Krox20::cre;Robo3lox/lox mutants, the DiI-labeled internuclear commissure is severely reduced. Some DiI-labeled fibers are still found on the contralateral side, but no cells are traced in the vicinity of the abducens nucleus. In Robo3−/− knockout (E and F), the commissure is almost completely absent. No cells are traced on the contralateral side. Scale bars represent 100 µm. (1.87 MB TIF) [file pbio.1000325.s005.tif]

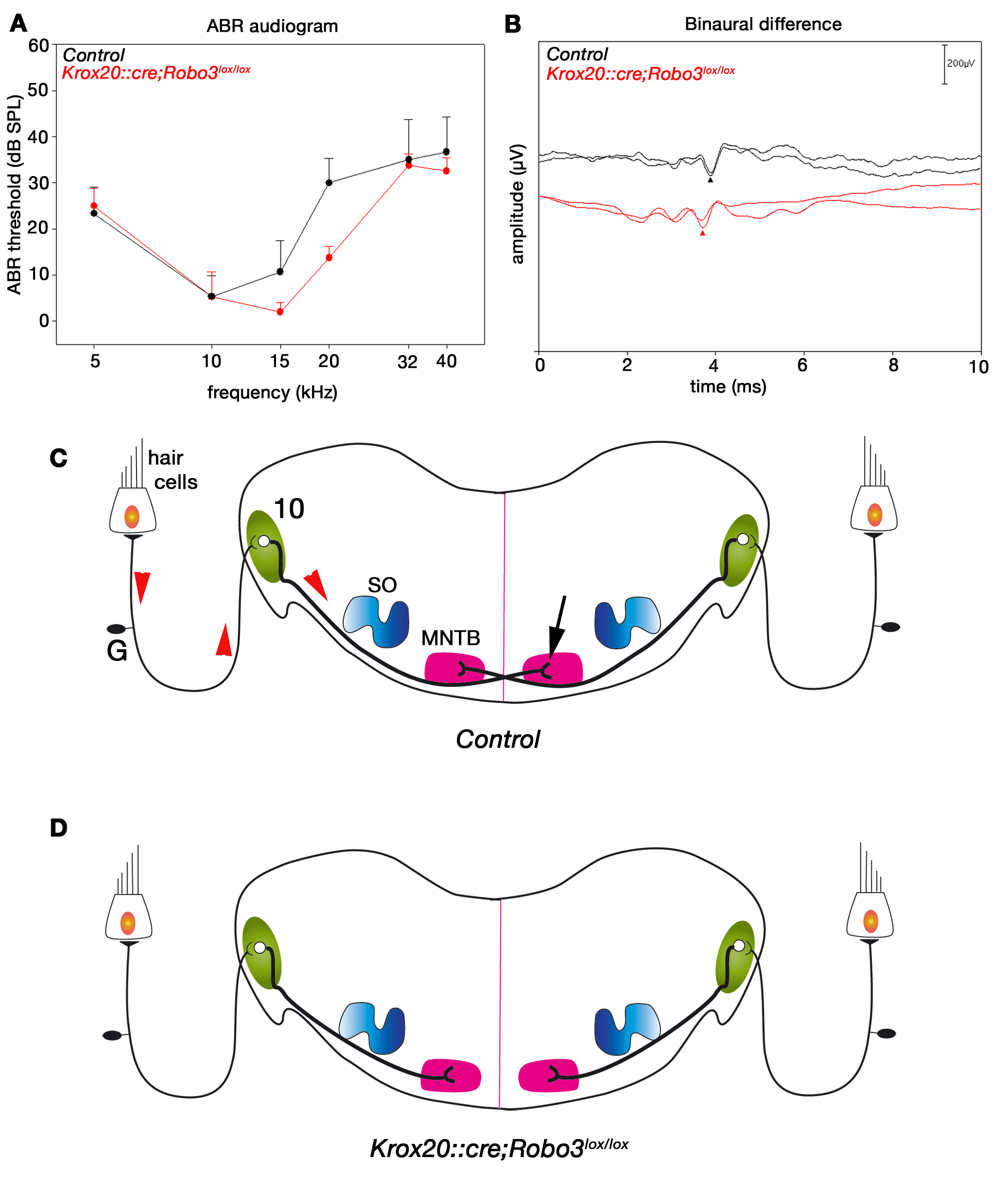

Supplement: Figure S6 — ABR thresholds and binaural difference in control and Krox20::cre;Robo3lox/lox mice. (A) The detection thresholds of ABR did not significantly differ in Krox20::cre;Robo3lox/lox versus control mice and remained in the normal range from 5–40 kHz. (B) The binaural-difference wave complex resulting from the fact that the late ABR waves in response to a diotic stimulus are smaller than the sum of waves in response to stimuli in either the right or the left side (see Materials and Methods), was still present in mutants, with similar amplitudes and latencies. It suggests that at least part of the functional coupling of left and right signals remained present in Krox20::cre;Robo3lox/lox mice, likely in relation to commissural neurons outside r3 and r5. (C and D) Schematic representation of the auditory pathway in control (C) and Krox20::cre;Robo3lox/lox mice (D). (G) Auditory inputs (red arrowheads) from the hair cells are transmitted to neurons of the spiral ganglion that project ipsilaterally into the brainstem on globular bushy cells in the anterior part of the ventral cochlear nucleus (aVCN). In controls, these cells send large-diameter axons to the contralateral medial nucleus of the trapezoid body (MNTB), forming calyces of Held synapses (arrow). MNTB neurons then project to the lateral superior olive (SO). In Krox20::cre;Robo3lox/lox mice, globular bushy cell axons only project to the ipsilateral MNTB but still form calyces. (C) is adapted from [4] (0.63 MB TIF) [file pbio.1000325.s006.tif]

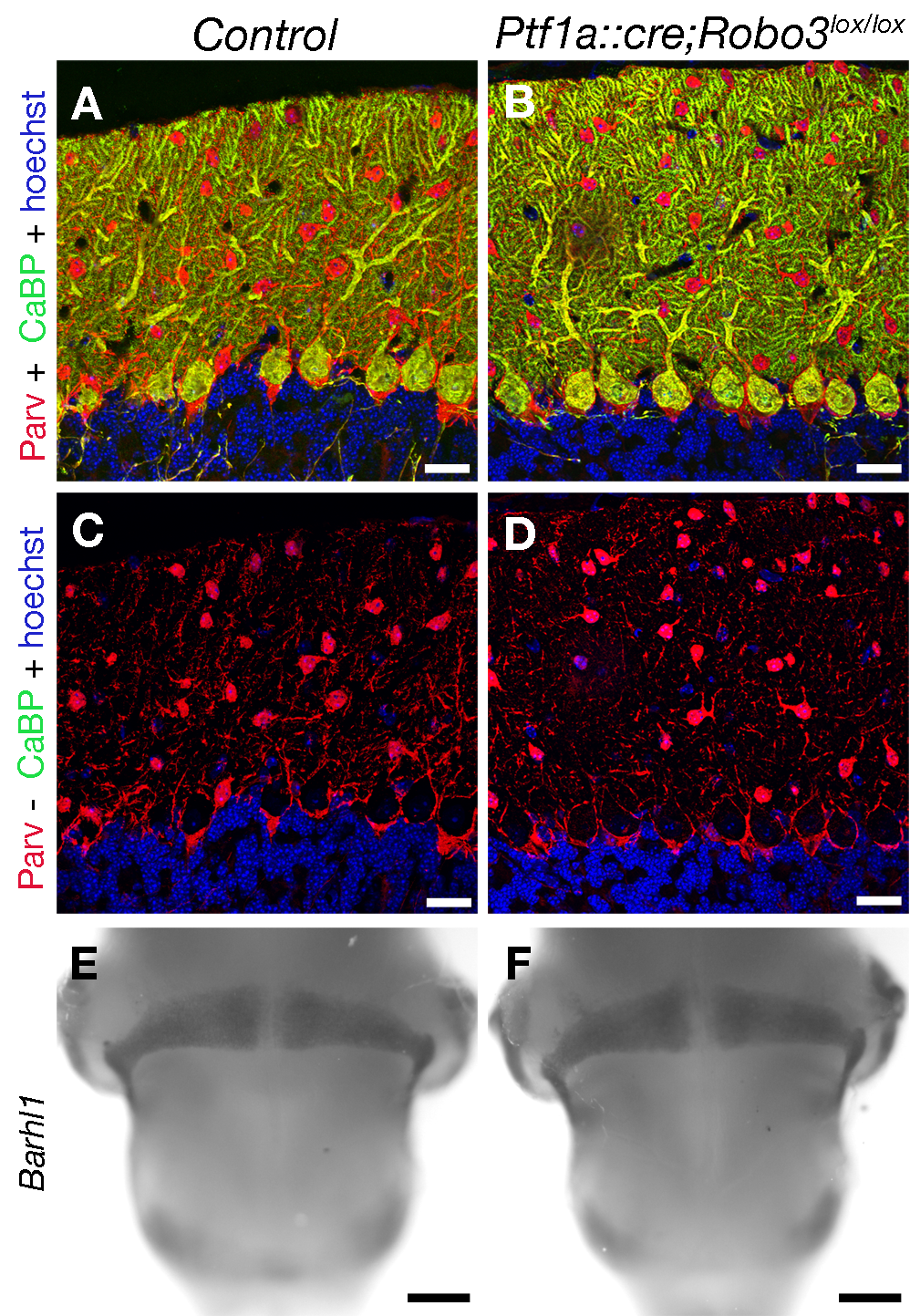

Supplement: Figure S7 — Normal cerebellar cortex and pontine nuclei in Ptf1a::cre;Robo3lox/lox mice (A–D) Sagittal sections of the cerebellar cortex of P32 control (A and C) and Ptf1a::cre;Robo3lox/lox mice (B and D) labeled with antibodies against parvalbumin (Parv) and calbindin (CaBP) and counterstained with Hoechst. Purkinje cells coexpress the two proteins, whereas molecular layer interneurons only express parvalbumin. (C and D) were obtained by subtraction of the calbindin channel (green) from the parvalbumin channel (red). The morphology of Purkinje cells and the density of molecular layer interneurons are similar. (E and F) show a ventral view of whole-mount hindbrain of E15 embryos hybridized with Barhl1 riboprobe. The stream of migrating pontine neurons (arrowheads) is comparable. Scale bars represent 25 µm, except in (E and F), where they indicate 500 µm (2.25 MB TIF) [file pbio.1000325.s007.tif]

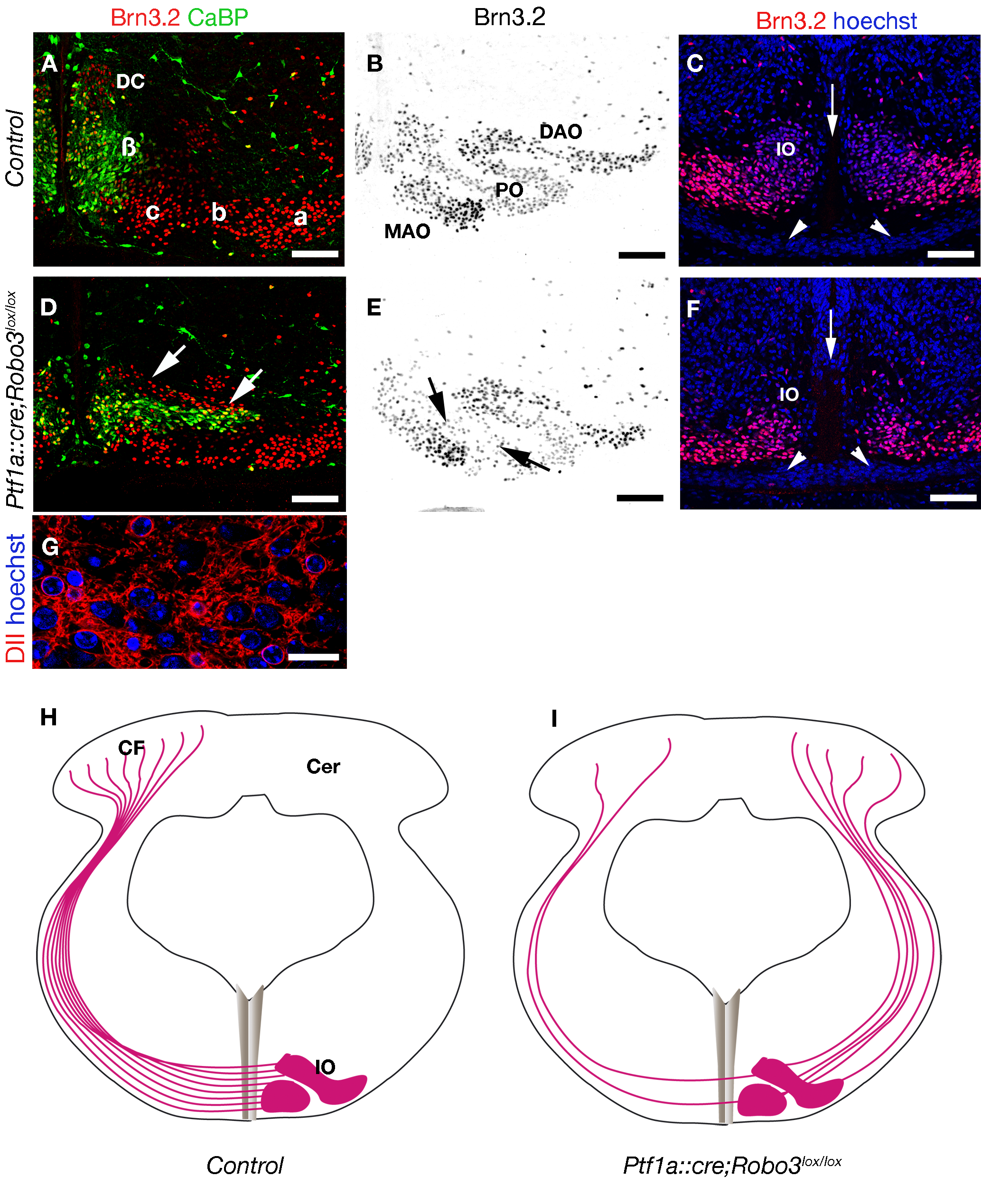

Supplement: Figure S8 — Phenotype of inferior olivary neurons in Ptf1a::cre;Robo3lox/lox mice. (A–F) Coronal sections of P0 (A, B, D, and E) and E13.5 (C and F) hindbrain at the level of the inferior olive labeled with Brn3.2 (A–F) and calbindin (A and D). The structure of the inferior olivary nucleus is disorganized in Ptf1a::cre;Robo3lox/lox mice (compare [A and B] with [D and E]), and many of its subdivisions have an abnormal shape. The arrows in (D) show the position of dorsal cap of Kooy (DC in [A]) and the β-nucleus (β in [A]) neurons, and the arrows in (E) indicate the disorganized principal olive (PO in [B]). (C and F) Brn3.2+ IO neurons do not cross the midline (arrow) in either control (C) or Ptf1a::cre;Robo3lox/lox (F) embryos. The arrowheads point to migrating LRN neurons. (G) is a 1.16-µm-thick confocal image of DiI-labeled IO neurons in control P0 mouse with Hoechst counterstaining. (H and I) Schematic representation of the olivocerebellar projection in control (H) and Ptf1a::cre;Robo3lox/lox mice (I). In control, all IO neurons project across the ventral midline to the contralateral cerebellum (Cer) where their terminal arborization, the climbing fibers (CF), synapse on Purkinje cells. In Ptf1a::cre;Robo3lox/lox mice, most IO axons project into the ipsilateral cerebellar cortex. Scale bars represent 100 µm, except in (G), where it indicates 20 µm a, b, and c indicate the subnuclei a, b, and c, respectively, of the MAO. DAO, dorsal accessory olive; DC, Dorsal Cap of Kooy; MAO, medial accessory olive. (3.31 MB DOC) [file pbio.1000325.s008.tif]

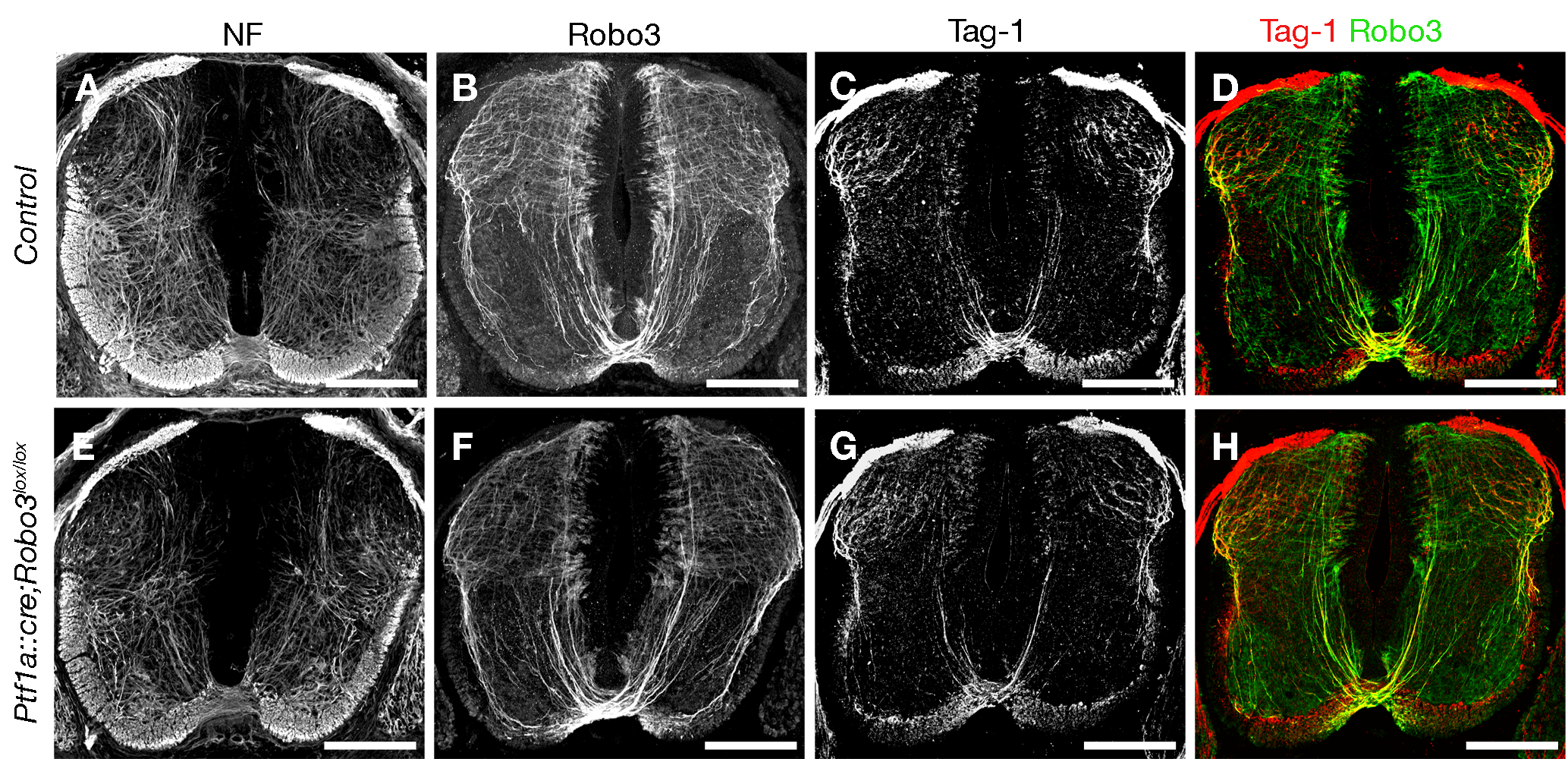

Supplement: Figure S9 — Normal spinal cord commissures in Ptf1a::cre;Robo3lox/lox embryos. (A–H) are coronal sections of the spinal cord of E13 embryos immunolabeled with neurofilament and Robo3 (A, B, E, and F) or TAG-1 and Robo3 (C, D, G, and H). Commissures are not reduced and still express Robo3 in Ptf1a::cre;Robo3lox/lox mice. Scale bars represent 200 µm. (2.74 MB TIF) [file pbio.1000325.s009.tif]
