# Supplementary material for: Genetic Dissection of the Function of Hindbrain Axonal Commissures
Source: PLoS Biol. 2010 Mar 9;8(3):e1000325. doi: 10.1371/journal.pbio.1000325 (PMC2834709; doi:10.1371/journal.pbio.1000325)
Supplement: Table S1 — (0.44 MB PDF) [file pbio.1000325.s010.pdf]

BONFERRONI-CORRECTED $\alpha = 0.0012$

HORIZONTAL EYE MOVEMENTS

	FREQ	Control AV	SD	SEM	n	Krox20::Cre;Robo3$^{lox/lox}$ AV	SD	SEM	n	REPEATED MEASURES ANOVA	ONE-WAY ANOVA	KRUSKAL-WALLIS
OKR	0.1	1.00	0.09	0.04	6	0.66	0.17	0.07	4		0.0065	0.0105
FIXED	0.2	0.99	0.06	0.03	6	0.71	0.15	0.06	4		0.0068	0.0105
AMPLITUDE	0.4	0.85	0.08	0.04	5	0.66	0.18	0.08	4	**0.043**	0.0814	0.0881
	0.6	0.61	0.05	0.02	6	0.52	0.20	0.08	4		0.4338	0.6698
	0.8	0.38	0.04	0.02	6	0.37	0.14	0.06	4		0.9364	1.0000
	1	0.32	0.01	0.01	6	0.29	0.13	0.05	4		0.6557	1.0000
VVOR	0.1	1.02	0.06	0.03	6	0.69	0.12	0.05	4		0.0012	0.0105
FIXED	0.2	1.02	0.06	0.03	6	0.80	0.13	0.05	4		0.0106	0.0105
AMPLITUDE	0.4	0.98	0.05	0.02	6	0.77	0.09	0.04	4	**0.000**	0.0025	0.0105
	0.6	1.08	0.03	0.02	6	0.79	0.14	0.06	4		0.0038	0.0105
	0.8	1.05	0.06	0.03	6	0.76	0.15	0.06	4		0.0066	0.0105
	1	1.08	0.05	0.02	6	0.77	0.18	0.07	4		0.0128	0.0330
VOR	0.1	0.19	0.06	0.03	2	0.05	0.03	0.02	3		0.0552	0.0833
FIXED	0.2	0.32	0.18	0.11	5	0.06	0.07	0.03	3		0.0250	0.0526
AMPLITUDE	0.4	0.36	0.12	0.07	5	0.06	0.08	0.03	3	**0.001**	0.0049	0.0253
	0.6	0.53	0.04	0.02	6	0.15	0.09	0.04	3		0.0003*	0.0201
	0.8	0.72	0.09	0.05	6	0.17	0.13	0.05	3		0.0004*	0.0201
	1	0.81	0.03	0.02	6	0.24	0.15	0.06	3		0.0005*	0.0201
OKR	0.05	0.98	0.05	0.02	6	0.30	0.16	0.06	4		0.0000*	0.0105
FIXED	0.1	0.98	0.05	0.02	6	0.46	0.12	0.05	4		0.0000*	0.0105
VELOCITY	0.2	0.92	0.06	0.03	6	0.61	0.14	0.06	4	**0.004**	0.0043	0.0105
	0.4	0.88	0.10	0.05	6	0.76	0.16	0.07	4		0.2211	0.2008
	0.8	0.84	0.11	0.06	6	0.72	0.23	0.09	4		0.3985	0.6698
	1.6	0.76	0.12	0.06	5	0.68	0.22	0.10	4		0.5365	0.6242

VERTICAL EYE MOVEMENTS

	FREQ	Control AV	SD	SEM	n	Krox20::Cre;Robo3$^{lox/lox}$ AV	SD	SEM	n	REPEATED MEASURES ANOVA	ONE-WAY ANOVA	KRUSKAL-WALLIS
OKR	0.1	0.81	0.18	0.07	6	0.91	0.04	0.02	3		0.3510	0.7963
FIXED	0.2	0.70	0.15	0.06	6	0.90	0.05	0.03	3		0.0682	0.0389
AMPLITUDE	0.4	0.48	0.11	0.05	6	0.46	0.05	0.03	3	**0.399**	0.8688	0.7963
	0.6	0.23	0.03	0.01	6	0.22	0.04	0.02	3		0.9209	0.7963
	0.8	0.13	0.02	0.01	6	0.11	0.04	0.02	4		0.2997	0.2864
	1	0.13	0.01	0.00	6	0.10	0.02	0.01	4		0.0111	0.0330
VVOR	0.1	0.87	0.11	0.05	6	0.89	0.05	0.03	4		0.6923	1.0000
FIXED	0.2	0.87	0.18	0.07	6	0.89	0.05	0.03	4		0.7638	1.0000
AMPLITUDE	0.4	0.92	0.11	0.04	6	0.89	0.07	0.03	4	**0.854**	0.6868	0.5224
	0.6	0.92	0.19	0.08	6	0.93	0.06	0.03	3		0.9636	0.6056
	0.8	0.90	0.10	0.04	6	0.94	0.04	0.02	4		0.4999	0.5224
	1	0.82	0.18	0.07	6	0.90	0.02	0.01	3		0.5192	0.4386
VOR	0.1	0.27	0.17	0.12	2	0.34	0.03	0.02	4		0.4412	1.0000
FIXED	0.2	0.42	0.18	0.08	5	0.49	0.13	0.07	4		0.5762	0.8065
AMPLITUDE	0.4	0.50	0.21	0.09	5	0.47	0.07	0.04	4	**0.584**	0.7909	0.8065
	0.6	0.49	0.17	0.08	5	0.61	0.15	0.08	4		0.3062	0.3272
	0.8	0.65	0.17	0.08	5	0.70	0.12	0.06	4		0.6215	0.6242
	1	0.57	0.22	0.10	5	0.72	0.13	0.06	4		0.3029	0.4624

* significantly different from corrected α

Note that the n's for 0.1Hz VOR data are very low; this is due to the fact that the VOR is too small to analyse at such low frequency.
