# Supplementary material for: Genetic Dissection of the Function of Hindbrain Axonal Commissures
Source: PLoS Biol. 2010 Mar 9;8(3):e1000325. doi: 10.1371/journal.pbio.1000325 (PMC2834709; doi:10.1371/journal.pbio.1000325)
Supplement: Table S2 — (1.59 MB PDF) [file pbio.1000325.s011.pdf]

Steptime

Descriptive statistics

Genotype	Mean Steptime	Std. Deviation	Std. Error of Mean
**Session1**			
Lurcher	655.86	199.38	66.46
Lurcher WT	270.62	143.97	71.99
ptf1a::cre;Robo3lox/lox	1165.90	723.33	295.29
*ptf1a::cre;Robo3lox/lox*WT	205.25	20.15	14.25
**Session2**			
Lurcher	617.02	151.28	50.43
Lurcher WT	251.00	103.91	51.96
ptf1a::cre;Robo3lox/lox	1153.80	471.01	192.29
*ptf1a::cre;Robo3lox/lox*WT	288.75	10.96	7.75
**Session3**			
Lurcher	552.21	149.30	49.76
Lurcher WT	257.12	148.73	74.36
ptf1a::cre;Robo3lox/lox	1341.30	486.15	198.29
*ptf1a::cre;Robo3lox/lox*WT	256.25	4.60	3.25

Multiple Comparisons

Tukey HSD		Mean Difference	Std. Error	Sig.
Lurcher	*Lurcher* WT	348.78	148.55	0.126
	ptf1a::cre;Robo3lox/lox	-611.94	130.29	0.001
	*ptf1a::cre;Robo3lox/lox*WT	358.28	193.25	0.284
Lurcher WT	*Lurcher*	-348.76	148.55	0.126
	ptf1a::cre;Robo3lox/lox	-960.72	159.57	0.000
	*ptf1a::cre;Robo3lox/lox*WT	9.5	214.08	1.000
ptf1a::cre; Robo3lox/lox	*Lurcher*	611.94	130.29	0.001
	Lurcher WT	960.72	159.57	0.000
	ptf1a::cre;Robo3lox/loxWT	970.22	201.84	0.001
*ptf1a::cre; Robo3lox/lox*WT	*Lurcher*	-358.28	193.25	0.284
	Lurcher WT	-9.5	214.08	1.000
	ptf1a::cre;Robo3lox/lox	-970.22	201.84	0.001

Tests of Between-Subjects Effects

Source	df	Mean Square	F	Sig.
Intercept	1	1.60E+07	87.059	0.000
Genotype	3	2874259.74	15.679	0.000

Percentage of good runs

Descriptive statistics

Genotype	Mean %	Std. Deviation	Std. Error of Mean
**Session1**			
Lurcher	85.16	6.21	2.07
Lurcher WT	88.20	3.52	1.76
ptf1a::cre;Robo3lox/lox	37.03	23.40	9.55
*ptf1a::cre;Robo3lox/lox*WT	85.07	7.88	3.94
**Session2**			
Lurcher	84.88	4.83	1.61
Lurcher WT	92.35	7.66	3.82
ptf1a::cre;Robo3lox/lox	30.83	19.60	8.00
*ptf1a::cre;Robo3lox/lox*WT	88.17	7.04	3.52
**Session3**			
Lurcher	85.80	9.34	3.11
Lurcher WT	96.50	2.68	1.34
ptf1a::cre;Robo3lox/lox	27.32	12.15	4.96
*ptf1a::cre;Robo3lox/lox*WT	84.37	8.13	4.06

Kruskal-Wallis Test

	S1percentage	S2percentage	S3percentage
Chi-Square	12.158	14.347	17.141
df	3	3	3
Asymp. Sig.	0.007	0.002	0.001

Mann-Whitney U test; Bonferroni-corrected α=0.004167

Lurcher* vs *ptf1a::cre;Robo3lox/lox

Session nr	Exact Sig.
1	0.001
2	0.000
3	0.000

***Lurcher* vs *Lurcher* WT**

Session nr	Exact Sig.
1	0.414
2	0.106
3	0.011

***ptf1a::cre;Robo3lox/lox* vs *ptf1a::cre;Robo3lox/lox*WT**

Session nr	Exact Sig.
1	0.038
2	0.010
3	0.010

Lurcher* WT vs *ptf1a::cre;Robo3lox/loxWT

Session nr	Exact Sig.
1	0.486
2	0.486
3	0.029
