# Supplementary material for: Genetic Dissection of the Function of Hindbrain Axonal Commissures
Source: PLoS Biol. 2010 Mar 9;8(3):e1000325. doi: 10.1371/journal.pbio.1000325 (PMC2834709; doi:10.1371/journal.pbio.1000325)
Supplement: Table S3 — (0.02 MB DOC) [file pbio.1000325.s012.doc]

| Cre | 5'-GCCAGATTACGTATATCCTGGCAGC-3' | 5'-ATCGCCAGGCGTTTTCTGAGCATAC-3' |
| --- | --- | --- |
| Lox (not excised) | primer n°1692  5'-CCAAGGAAAAACTTGAGGTTGCAGCTAG-3' | primer n°1694  5'-GATTAGGGGAGGTGAGACATAGGG-3' |
| Lox  (excision) | primer n°1692  5'-CCAAGGAAAAACTTGAGGTTGCAGCTAG-3' | primer n°1697  5'-CTCCTCCTGGTTACCTCTGTAAAAGATTC-3' |
| GFP | 5'-GAGGGCGATGCCACCTACGGCAAG-3' | 5'-CTCAGGGCGGACTGGGTGCTCAGG-3' |
| Robo3KO (mutant) | 5'-GATCTCTCGTGGGATCATTG-3' | 5'-TACCAGCTACTTCCAGAGAG-3' |
| Robo3KO (WT) | 5'-CCAACATCGAGTGGTACAAG-3' | 5'-TACCAGCTACTTCCAGAGAG-3' |

# Table S3. Primer pairs used for mouse genotyping.
