# Supplementary material for: Genetic Dissection of the Function of Hindbrain Axonal Commissures
Source: PLoS Biol. 2010 Mar 9;8(3):e1000325. doi: 10.1371/journal.pbio.1000325 (PMC2834709; doi:10.1371/journal.pbio.1000325)
Supplement: Text S1 — Supplemental Methods. Eye movement and ABR recordings. (0.02 MB DOC) [file pbio.1000325.s013.doc]

**Methods**

**Eye movement recordings**

All mice for eye movement recordings were between 2 and 6 months of age and were surgically prepared for experiments under general anesthesia of a mixture of isoflurane (Rhodia Organique Fine Ltd, Bristol, UK) and oxygen. A construct consisting of two nuts was attached to the frontal and parietal bones using Optibond prime and adhesive (Kerr, Bioggio, Switzerland) and Charisma (Heraeus Kulzer, Armonk, NY, USA). After a recovery period of 5 days the mouse was placed in a restrainer, with its head bolted to a bar. The restrainer was fixed onto the centre of the turntable. A cylindrical screen (diameter 61 cm) with a random-dotted pattern (each element 2°) surrounded the turntable (diameter 59 cm). The OKR and VOR were evoked by rotating the surrounding screen and turntable, respectively, with a fixed peak amplitude of 5° at different frequencies (0.1 – 1.0 Hz) and with a fixed peak velocity of 8°/s (at 0.05 – 1.6Hz). The surrounding screen and the turntable were driven independently by AC servo-motors (Harmonic Drive AG, The Netherlands). The table and drum position signal were measured by potentiometers, filtered, digitized (CED Limited, UK), and stored on a computer. A CCD camera was fixed to the turntable in order to monitor the mouse's eye. The eye movements were recorded at 240 Hz using the eye-tracking device of ISCAN (Iscan Inc.). Video calibrations and subsequent eye movement computations were performed as described previously. Off-line analysis of eye movements was performed in Matlab (MathWorks, Natick, MA). The gain and the phase of the eye movements were determined by fitting sine functions to the slow-phase eye velocity traces. Gain was computed as the ratio of eye velocity to stimulus velocity, whereas phase was expressed as the difference (in degrees) between the eye velocity and stimulus velocity traces.

**Recordings of auditory brainstem-evoked responses (ABR)**

ABRs were used in three ways, for measuring auditory thresholds in the 5-40 kHz range, for assessing neural conduction at sound levels well above threshold and for ascertaining the presence of binaural interactions. ABR thresholds were determined by visual inspection of waves I-IV and defined, at every frequency of interest, as the lowest stimulus level such that at least one ABR wave remained present above noise floor [1]. Neural conduction was evaluated by measuring the latencies and amplitudes of the first four waves following stimulus onset in ipsilateral and contralateral recordings [1]. The sound level for evaluating neural conduction was set at 60 dB SPL, i.e. loud enough for all characteristic waves to be evident yet soft enough to avoid crosstalk between ears due to bone-conducted sound vibrations from the stimulated to the contralateral ear. Next, the difference between the ABR waveform in response to a diotic 60 dB SPL click and the sum of ABR waveforms in response to the same click applied either ipsi- or contralaterally, was mathematically extracted. In normal subjects, this procedure is known to generate a so-called binaural difference reflecting a functional coupling of left and right signals possibly including localization processes [2].

The result of synchronously averaging the electroencephalographic activity in the presence of repeated brief acoustic stimulations is an ABR waveform [3], characteristically made of a sequence of peaks numbered I, II, III and IV at increasing delay relative to stimulus onset. The presence, latency and size of ABR waves correlate with gross features of auditory pathways across the hindbrain. On the ipsilateral side, it is established that the first ABR peak, numbered I, reflects the activity of spiral ganglion cells. Later waves II, III and IV are ascribed to the sequential activation of more and more central neural generators.

**References**

1. Delmaghani S, del Castillo FJ, Michel V, Leibovici M, Aghaie A, et al. (2006) Mutations in the gene encoding pejvakin, a newly identified protein of the afferent auditory pathway, cause DFNB59 auditory neuropathy. Nat Genet 38: 770-778.

2. Riedel H, Kollmeier B (2006) Interaural delay-dependent changes in the binaural difference potential of the human auditory brain stem response. Hear Res 218: 5-19.

3. Jewett DL, Williston JS (1971) Auditory-evoked far fields averaged from the scalp of humans. Brain 94: 681-696.

4. Schneggenburger R, Forsythe ID (2006) The calyx of Held. Cell Tissue Res 326: 311-337.
